# Endothelial FAT1 inhibits angiogenesis by controlling YAP/TAZ protein degradation via E3 ligase MIB2

**DOI:** 10.1038/s41467-023-37671-x

**Published:** 2023-04-08

**Authors:** Rui Li, Jingchen Shao, Young-June Jin, Haruya Kawase, Yu Ting Ong, Kerstin Troidl, Qi Quan, Lei Wang, Remy Bonnavion, Astrid Wietelmann, Francoise Helmbacher, Michael Potente, Johannes Graumann, Nina Wettschureck, Stefan Offermanns

**Affiliations:** 1 Max Planck Institute for Heart and Lung Research, Department of Pharmacology, Ludwigstr. 43, 61231 Bad Nauheim, Germany; 2 Max Planck Institute for Heart and Lung Research, Angiogenesis & Metabolism Laboratory, Ludwigstr. 43, 61231 Bad Nauheim, Germany; 3 Department of Vascular and Endovascular Surgery, Cardiovascular Surgery Clinic, University Hospital Frankfurt and Wolfgang Goethe University Frankfurt, Frankfurt, Germany; 4 Max Planck Institute for Heart and Lung Research, Small Animal Imaging Service Group, Ludwigstr. 43, 61231 Bad Nauheim, Germany; 5 Aix Marseille Université, CNRS, IBDM UMR 7288, Parc Scientifique de Luminy, Case 907, 13288 Marseille, France; 6 Max Planck Institute for Heart and Lung Research, Biomolecular Mass Spectrometry Service Group, Ludwigstr. 43, 61231 Bad Nauheim, Germany; 7 Center for Molecular Medicine, Goethe University Frankfurt, Theodor-Stern-Kai 7, 60590 Frankfurt, Germany; 8 Cardiopulmonary Institute, Bad Nauheim, Germany; 9 German Center for Cardiovascular Research, Partner Site Frankfurt, Bad Nauheim, Germany; 10 Present Address: Berlin Institute of Health at Charité—Universitätsmedizin Berlin, and Max Delbrück Center for Molecular Medicine in the Helmholtz Association, Berlin, Germany; 11 Present Address: Institute of Translational Proteomics, Department of Medicine, Philipps-University Marburg, Karl-von-Frisch-Str. 2, 35043 Marburg, Germany

**Keywords:** Cell growth, Cell signalling, Angiogenesis

## Abstract

Activation of endothelial YAP/TAZ signaling is crucial for physiological and pathological angiogenesis. The mechanisms of endothelial YAP/TAZ regulation are, however, incompletely understood. Here we report that the protocadherin FAT1 acts as a critical upstream regulator of endothelial YAP/TAZ which limits the activity of these transcriptional cofactors during developmental and tumor angiogenesis by promoting their degradation. We show that loss of endothelial FAT1 results in increased endothelial cell proliferation in vitro and in various angiogenesis models in vivo. This effect is due to perturbed YAP/TAZ protein degradation, leading to increased YAP/TAZ protein levels and expression of canonical YAP/TAZ target genes. We identify the E3 ubiquitin ligase Mind Bomb-2 (MIB2) as a FAT1-interacting protein mediating FAT1-induced YAP/TAZ ubiquitination and degradation. Loss of MIB2 expression in endothelial cells in vitro and in vivo recapitulates the effects of FAT1 depletion and causes decreased YAP/TAZ degradation and increased YAP/TAZ signaling. Our data identify a pivotal mechanism of YAP/TAZ regulation involving FAT1 and its associated E3 ligase MIB2, which is essential for YAP/TAZ-dependent angiogenesis.

## Abstract (web summary)

The authors report that endothelial protocadherin FAT1 inhibits endothelial proliferation and angiogenesis by promoting degradation of the transcriptional cofactors YAP and TAZ by direct interaction with the E3 ubiquitin ligase Mind Bomb-2 (MIB2).

## Introduction

The proto-cadherin Fat has originally been described in *Drosophila* to reduce proliferation in a variety of tissues and to regulate critical developmental processes^1,2^. In the mammalian system, there is one Fat homolog, FAT4, and three Fat-like homologs, FAT1-3^1,2^. FAT1 has been shown to be involved in tissue morphogenesis and vascular smooth muscle remodeling^3–8^. Mutations in *FAT1* have been found in various tumors where it functions as a tumor suppressor gene^9–13^. FAT1 has been shown to negatively regulate YAP and TAZ in zebrafish and mammals^11,14,15^, and loss of FAT1 function in tumor cells results in YAP/TAZ activation^10,13^ through incompletely understood mechanisms.

YAP and TAZ are transcriptional cofactors, which shuttle between the cytoplasm and the nucleus. By integrating different cellular signaling pathways, these proteins regulate cellular functions such as cell proliferation, differentiation and survival^16–19^. In the nucleus, YAP/TAZ can activate or repress numerous genes by interacting with different transcription factors, of which TEAD family members are the best characterized^20–23^. In endothelial cells, YAP/TAZ regulate sprouting angiogenesis during development and tumor growth^24–30^, vascular barrier maturation and maintenance^24,29^ as well as flow-dependent regulation of vessel maintenance and progression of atherosclerosis^31–33^.

YAP/TAZ activity is controlled by several upstream signaling pathways. Mechanical cues generated by extracellular matrix stiffness and cytoskeletal tension promote nuclear translocation and activation of YAP/TAZ through modulation of filamentous-actin (F-actin) structures, via poorly understood molecular mechanisms^18,34–36^. In contrast, the Hippo signaling pathway is a central negative regulator of YAP/TAZ in *Drosophila* and mammals^17,37^. In the Hippo signaling cascade, MST1/2 kinases phosphorylate and activate LATS1/2 kinases, which directly phosphorylate YAP and TAZ, thereby promoting their retention and degradation in the cytosol^17,19^.

In the present study, we discovered that endothelial FAT1 promotes basal YAP/TAZ protein turnover by interacting with the E3 ligase MIB2, leading to their subsequent ubiquitin-dependent degradation. Loss of endothelial FAT1 or MIB2 stabilized YAP/TAZ protein levels and increased YAP/TAZ transcriptional activity, resulting in increased endothelial proliferation in vitro and in vivo. Our work identifies FAT1 as an essential suppressor of endothelial proliferation and shows that YAP/TAZ degradation is the critical mechanism through which FAT1 signals in endothelial cells.

## Results

### Endothelial FAT1 controls cell proliferation

**Fig. 1:**
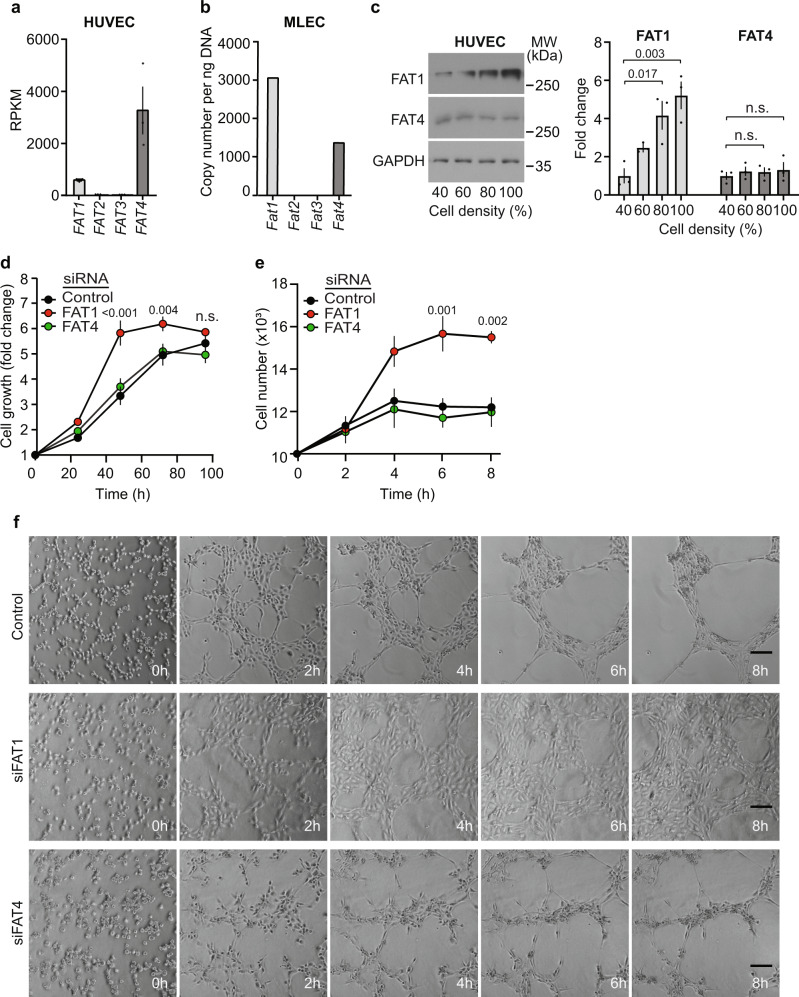
Loss of endothelial FAT1 increases cell proliferation. **a**, **b** Expression of FAT1-4 in HUVECs (**a**) and mouse lung endothelial cells (MLECs) (**b**) determined by RNA-sequencing (**a**; *n* = 3 independently performed experiments) and RT-qPCR (**b**). **c** HUVECs were cultured and lysed at the indicated densities. Thereafter, protein levels of FAT1 and FAT4 were analyzed by Western blotting. GAPDH served as a loading control. Shown is a representative experiment and the statistical evaluation of a densitometric analysis (*n* = 3 independent experiments). **d** HUVECs were transfected with control siRNA or siRNA directed against *FAT1* or *FAT4* and cell proliferation was determined using a colorimetric cell proliferation assay 24, 48, 72 and 96 h after seeding of cells. Results were expressed as the fold change relative to the cell numbers at 0 h (time of transfection) (*n* = 6 independent experiments). **e**, **f** HUVECs transfected with control siRNA or siRNA directed against *FAT1* or *FAT4* were seeded in matrigel for 3D culture. Cells were counted after matrigel digestion. Shown are the statistical evaluation (**e**; *n* = 3 independently performed experiments) as well as pictures taken directly after seeding and every 2 h afterwards (**f**). Bar length: 100 µm. Shown are mean values ± SEM. n.s. non-significant (one-way ANOVA and Tukey’s post hoc test (**c**) and two-way ANOVA and Bonferroni’s post hoc test (**d** and **e**)).

Expression analysis of human umbilical vein endothelial cells (HUVECs), mouse lung endothelial cells (MLECs) and several other endothelial cell types showed that FAT1 and FAT4 are the sole members of the FAT family of proto-cadherins expressed in endothelial cells (Fig. 1a, b and Supplementary Fig. 1a–c). When analyzing the expression of FAT1 and FAT4 in proliferating HUVECs and human umbilical artery endothelial cells (HUAECs), we found that FAT1 protein levels were relatively low during proliferation but increased with increasing endothelial cell density. In contrast, the protein levels of FAT4 remained unchanged (Fig. 1c and Supplementary Fig. 1d). The increase in FAT1 protein levels at a higher cell density was not due to increased transcription of the *FAT1* gene but to reduced FAT1 protein degradation (Supplementary Fig. 1e, f). To study the role of FAT1 and FAT4 in endothelial cells in vitro, we suppressed *FAT1* and *FAT4* expression by siRNA-mediated knock-down (Supplementary Fig. 1g, h) and found that knock-down of FAT1 significantly increased the proliferation of endothelial cells, which reached confluency much faster than control cells. In contrast, knock-down of FAT4 had no effect (Fig. 1d and Supplementary Fig. 1i). Comparable effects could be seen when HUVECs were cultured in matrigel to allow for three-dimensional growth and tube formation (Fig. 1e, f).

### Loss of endothelial FAT1 causes endothelial hyperproliferation in vivo

**Fig. 2:**
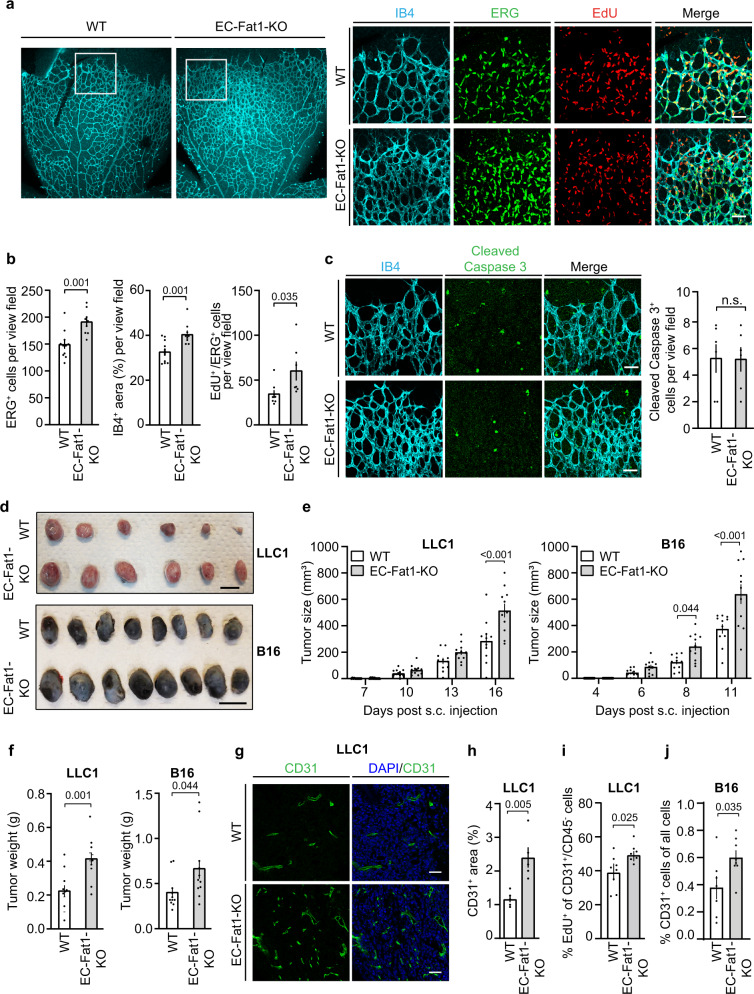
Loss of endothelial FAT1 results in increased postnatal and tumor angiogenesis in vivo. **a**–**c** Wild-type (WT) and EC-Fat1-KO newborns were treated at P1-3 with tamoxifen, and retinae were prepared at P6. Shown are representative photomicrographs of retinae stained for isolectin B4 (IB4), ERG and cleaved caspase 3 as well as for EdU after treatment for 6 h (**a**, **c**) and the statistical evaluation of staining for IB4 (*n* = 12 mice, WT; *n* = 9 mice, EC-Fat1-KO), for ERG (*n* = 11 mice, WT; *n* = 9 mice, EC-Fat1-KO), for EdU (*n* = 8 mice, WT; *n* = 7 mice, EC-Fat1-KO), and for cleaved caspase 3 (*n* = 6 mice) (**b**, **c**). Scale bar: 50 µm. **d**–**f** Tumor development after subcutaneous injection of Lewis lung carcinoma cells (LLC1) or B16 melanoma cells (B16) (**d**, **e**) and final tumor weight after 16 and 11 days, respectively (**f**) (*n* = 11 animals per group). Bar length (**d**): 1 cm. **g**, **h** Vascularization of LLC1 tumors was determined after staining with DAPI and an anti-CD31 antibody (**g**, exemplary photomicrograph; **h**, statistical evaluation) (*n* = 5 mice per group; 10–15 randomly selected sections were analyzed per mouse). Scale bar (**g**): 50 µm. **i** LLC1 tumor-bearing mice received EdU through their drinking water for 1 week before the end of the experiment and EdU-positive endothelial cells (Edu^+^/CD31^+^; CD45^−^) were analyzed by flow cytometry (*n* = 8 mice per group). **j** Mice subcutaneously injected with B16 tumor cells were sacrificed after 11 days, and the percentage of CD31-positive cells in tumors was analyzed by flow cytometry (*n* = 7 mice (WT); *n* = 8 mice (EC-Fat1-KO)). Data are presented as mean ± SEM. n.s. non-significant. Comparisons were performed using two-tailed unpaired *t*-test (**b**, **c**, **f**, **h**–**j**) or two-way ANOVA and Bonferroni’s post hoc test (**e**).

To test whether the anti-proliferative function of FAT1 in endothelial cells in vitro can also be seen under in vivo conditions, we generated tamoxifen-inducible endothelium-specific FAT1-deficient mice (Tek-CreERT2;*Fat1*^flox/flox^, herein referred to as EC-Fat1-KO). To study postnatal retinal angiogenesis, mice were treated with tamoxifen from postnatal day (P)1–P3, and retinae were analyzed at P6^38^. Endothelium-specific loss of FAT1 resulted in a significantly increased number of endothelial cells and a denser vasculature (Fig. 2a, b). The increase in number of endothelial cells was accompanied by increased incorporation of EdU whereas no difference in the number of cleaved caspase 3-positive cells could be observed (Fig. 2b, c). This indicates that FAT1 deficiency promotes endothelial cell proliferation in the developing retina.

We then studied the effect of endothelial FAT1 deficiency on tumor angiogenesis. In two syngeneic tumor models using B16 melanoma cells and Lewis lung carcinoma cells (LLC1), we observed a significantly increased tumor volume after subcutaneous injection of tumor cells in EC-Fat1-KO mice (Fig. 2d–f). Histological analysis of the tumors revealed an increased vessel area in tumors of EC-Fat1-KO mice (Fig. 2g, h), and flow cytometric analysis confirmed an increased number of endothelial cells with an increased proportion of EdU^+^ incorporation (Fig. 2i, j).

**Fig. 3:**
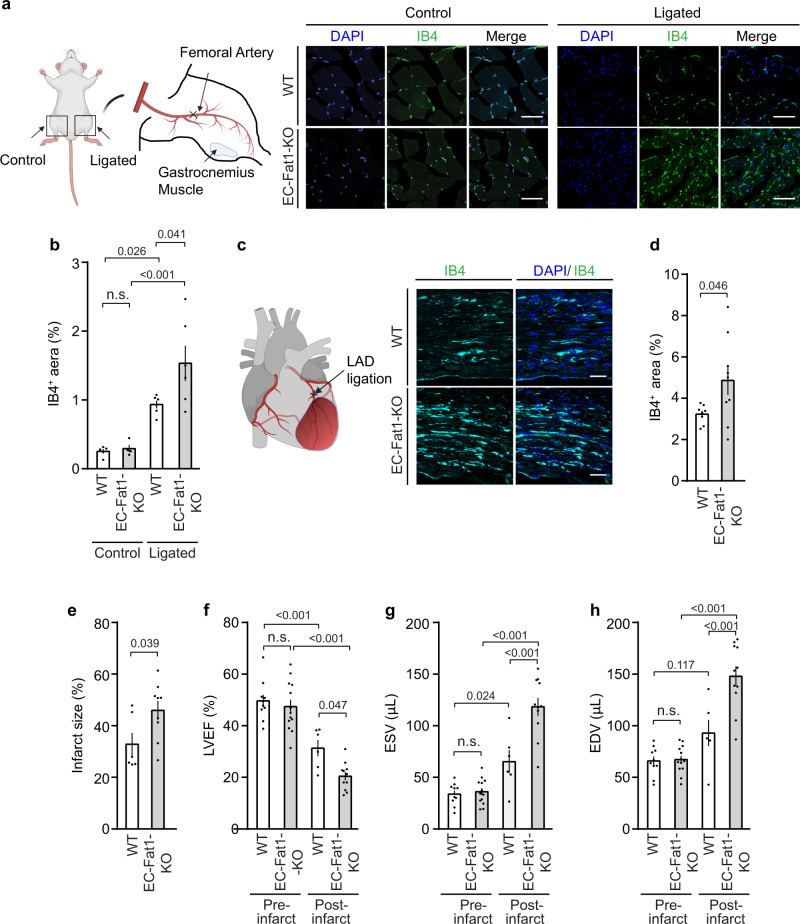
Loss of endothelial FAT1 increases ischemia-induced angiogenesis in vivo. **a**, **b** Hind-limb ischemia was induced by femoral artery ligation in wild-type and EC-Fat1-KO mice. Shown are representative photomicrographs of sections of the gastrocnemius muscle of the ligated hind limb (ligated) and of the unligated side (control) 14 days after the operation (*n* = 5 mice, WT unligated control; *n* = 4 mice, EC-FAT1-KO unligated control; *n* = 6 mice, WT and EC-Fat1-KO ligation). Shown are representative sections stained with DAPI and IB4 (**a**) and the statistical evaluation of the IB4^+^-area (**b**). Bar length (**a**): 50 µm. **c**–**h** Myocardial ischemia was induced by ligation of the left anterior descending coronary artery in wild-type (WT) and EC-Fat1-KO mice, and vascularization of the infarct zone was determined by IB4 staining (**c**, **d**) (*n* = 8 mice, WT; *n* = 9 mice, EC-Fat1-KO). Bar length (**c**): 50 µm. **e** Quantification of infarct size as determined by picrosirius red staining in hearts from control and EC-Fat1-KO mice after 14 days of acute myocardial infarction (*n* = 6 mice (WT); *n* = 9 mice (EC-Fat1-KO)). **f**–**h** Heart function was analyzed by magnetic resonance imaging of control and EC-Fat1-KO mice before and 14 days after acute myocardial infarction (*n* = 10 mice (WT pre-infarct); *n* = 13 mice (EC-Fat1-KO pre-infarct); *n* = 6 mice (WT post-infarct); *n* = 11 mice (EC-Fat1-KO post-infarct)). Shown are the left ventricular ejection fraction (LVEF) (**f**), the end systolic volume (ESV) (**g**) and the end diastolic volume (EDV) (**h**). All values are mean ± SEM. n.s. non-significant. Comparisons were performed using one-way ANOVA and Tukey’s post hoc test (**b**, **f**–**h**) or two-tailed unpaired *t*-test (**d**, **e**). Drawing displayed in (**a**) and (**c**) were created with BioRender.com.

In the adult organism, ischemia-induced neovascularization requires increased endothelial cell proliferation. To test whether ischemia-induced angiogenesis is also controlled by FAT1, we examined endothelial cell density in sections of the mouse gastrocnemius muscle 14 days after induction of hind limb ischemia. While wild-type mice showed the expected increase in endothelial cell density compared to the control side of operated animals, EC-Fat1-KO animals showed an even further increase in endothelial density (Fig. 3a, b). To determine whether a similar effect of endothelial FAT1 loss may also be seen in ischemia-induced angiogenesis in other vascular beds, we assessed endothelial cell density in the infarct zones 14 days after induction of acute myocardial infarction. We found an increased endothelial cell density in the infarct zone (Fig. 3c, d). However, increased endothelial cell density was not accompanied by improved cardiac function but rather by increased infarct size and reduced cardiac function 14 days after myocardial infarction (Fig. 3e–h), suggesting non-productive angiogenesis.

### YAP/TAZ mediate increased endothelial proliferation in the absence of FAT1

**Fig. 4:**
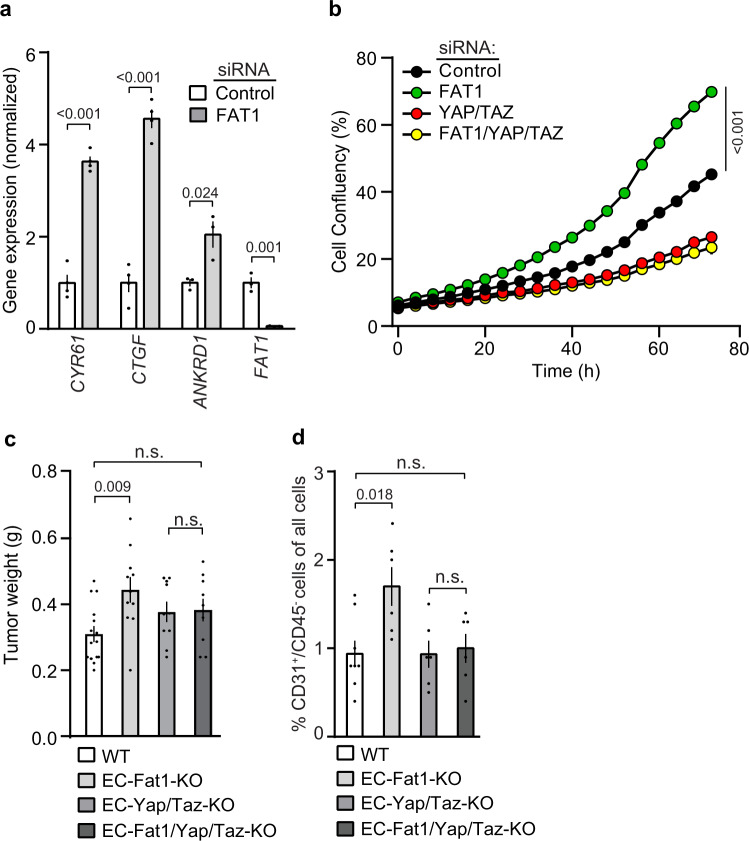
YAP/TAZ mediate increased endothelial proliferation in the absence of FAT1 in vitro and in vivo. **a** Expression of YAP/TAZ target genes in HUVECs after transfection with control siRNA or siRNA directed against *FAT1* (*n* = 3 (*ANKRD1*, *FAT1*) and *n* = 4 (*CYR61*, *CTGF*)) independent experiments; all data normalized to GAPDH and controls were set as 1. **b** Growth curve of HUVECs after siRNA-mediated knock-down of FAT1, YAP/TAZ or FAT1/YAP/TAZ. Cell growth was determined using a live-cell imaging system as described in the “Methods” section. The time of transfection was set as “0” (*n* = 4 independent experiments per group). **c**, **d** Tumor weight 16 days after subcutaneous injection of Lewis lung carcinoma cells (LLC1) in wild-type (WT), EC-Fat1-KO, EC-Yap/Taz-KO and EC-Fat1/Yap/Taz-KO mice (*n* = 15 mice (WT); *n* = 11 mice (EC-Fat1-KO); *n* = 10 mice (EC-Yap/Taz-KO and EC-Fat1/Yap/Taz-KO)) (**c**) and percentage of endothelial cells (CD31^+^CD45^−^) of all cells in LLC1 tumor samples of the indicated genotypes determined by flow cytometry (**d**) (*n* = 8 mice (WT); *n* = 6 mice (EC-Fat1-KO, EC-Yap/Taz-KO and EC-Fat1/Yap/Taz-KO)). All values are mean ± SEM. n.s. non-significant. Comparisons were performed using two-tailed unpaired *t*-test (**a**)) or one-way ANOVA and Tukey’s post hoc test (**c**, **d**) or two-way ANOVA and Bonferroni’s post hoc test (**b**).

To understand the underlying mechanisms of endothelial hyperproliferation in the absence of FAT1, we tested whether the expression of YAP/TAZ target genes was affected by suppression of *FAT1* expression in HUVECs. As shown in Fig. 4a, knock-down of FAT1 increased expression of bona fide YAP/TAZ targets such as *CYR61*, *CTGF* or *ANKRD1*, suggesting that loss of FAT1 resulted in increased YAP/TAZ activation. To test whether YAP and TAZ are involved in the increased proliferation response of endothelial cells after FAT1 suppression, we compared the effects of FAT1 knock-down alone with those elicited by the combined depletion of FAT1, YAP and TAZ. Figure 4b shows that the increased density of HUVECs after knock-down of FAT1 was abrogated in the absence of YAP and TAZ. To test whether this is also the case under in vivo conditions, we generated tamoxifen-inducible endothelium-specific FAT1/YAP/TAZ-triple-deficient mice (*Tek-CreERT2*;*Fat1*^flox/flox^;*Yap*^flox/flox^;*Taz*^flox/flox^, herein referred to as EC-Fat1/Yap/Taz-KO). The increased tumor weight and endothelial cell number seen in EC-Fat1-KO mice could not be observed when Yap and Taz were also eliminated in an endothelium-specific manner (Fig. 4c, d).

### Loss of FAT1 expression results in increased YAP and TAZ protein levels

**Fig. 5:**
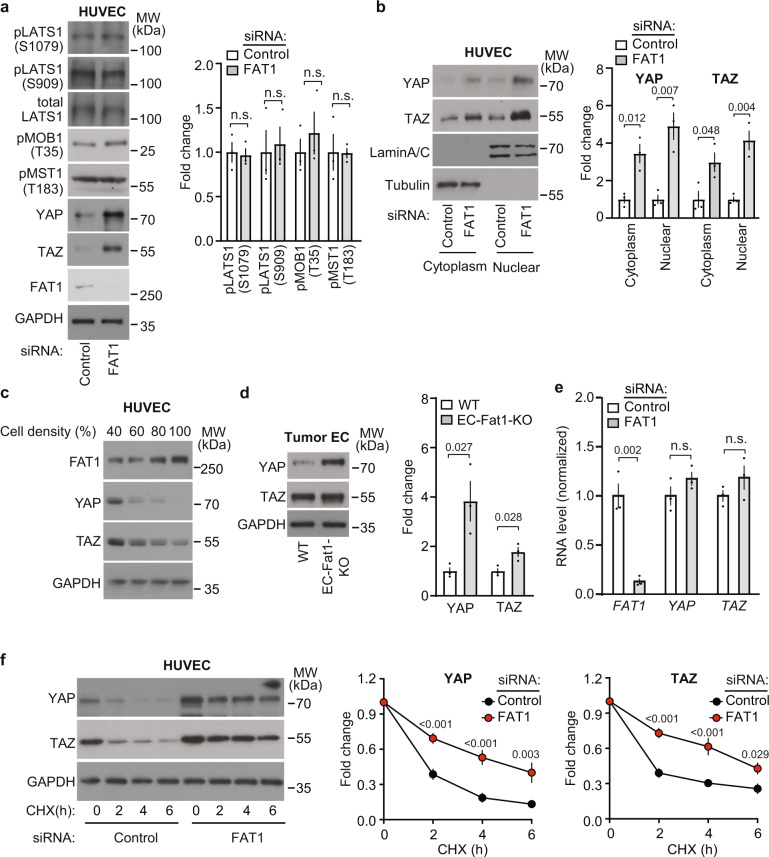
Loss of FAT1 expression results in increased YAP/TAZ protein stability in HUVECs. **a** Immunoblot analysis of total and phosphorylated Hippo pathway components in lysates of HUVECs transfected with control siRNA or siRNA directed against *FAT1*. Shown is a representative blot and the densitometric analysis (*n* = 3 independent experiments). **b** YAP/TAZ protein levels in the cytoplasmic and nuclear fraction of HUVECs transfected with control siRNA or siRNA directed against *FAT1*. LaminA/C and tubulin were used as markers for nuclear and cytoplasmic proteins. Shown are a representative blot and the statistical analysis (*n* = 3 independent experiments). **c** HUVECs were transfected with control siRNA or with siRNA directed against *FAT1* and were cultured and lyzed at the indicated cell densities. Thereafter, protein levels of FAT1, YAP and TAZ were analyzed by Western blotting (shown is a representative of three independently performed experiments). GAPDH served as a loading control. **d** YAP and TAZ protein levels in endothelial cells which were isolated from primary LLC1 tumors of control and EC-Fat1-KO mice. Shown is a representative blot and the statistical analysis (*n* = 3 mice). **e** YAP and TAZ as well as FAT1 mRNA levels in HUVECs after transfection with control siRNA or siRNA directed against *FAT1* (*n* = 3 independently performed experiments). **f** YAP and TAZ protein turnover in HUVECs transfected with control siRNA or siRNA directed against *FAT1* as determined by incubation of cells with 50 µg/ml of cycloheximide (CHX) for the indicated time periods. Shown is a representative immunoblot and the statistical evaluation (*n* = 4 (YAP) and *n* = 5 (TAZ) independent experiments). Data are presented as mean ± SEM. n.s. non-significant (two-tailed unpaired *t*-test (**a**, **b**, **d**, **e**) or two-way ANOVA and Bonferroni’s post hoc test (**f**)).

Since YAP/TAZ appear to mediate the increased endothelial proliferation in the absence of FAT1, we tested whether suppression of FAT1 expression has an effect on the Hippo pathway. siRNA-mediated knock-down of FAT1 did not change the phosphorylation state of MST1, LATS1 or MOB (Fig. 5a). This indicates that the Hippo kinase cascade is not involved in the observed regulation of YAP/TAZ by FAT1. When assessing YAP/TAZ protein levels in endothelial cells, we noticed that the levels of YAP and TAZ increased substantially after knock-down of FAT1 and correlated with an overproportionate accumulation of YAP and TAZ in the nucleus (Fig. 5b). Conversely, when FAT1 levels increased while endothelial cells became confluent (Fig. 1c), we observed a substantial decline in YAP/TAZ protein levels (Fig. 5c). Increased YAP and TAZ protein levels were also seen in endothelial cells under in vivo conditions when isolating endothelial cells from LLC1 tumors of EC-Fat1-KO animals (Fig. 5d). Knock-down of FAT1 had no effect on YAP and TAZ mRNA levels (Fig. 5e), indicating that the increased YAP/TAZ levels after FAT1 knock-down were not the result of increased YAP and TAZ transcription or mRNA stability. To test whether loss of FAT1 would affect YAP/TAZ protein degradation in endothelial cells, we determined YAP and TAZ protein levels in the absence and presence of cycloheximide. In control cells, cycloheximide-induced blockade of protein synthesis resulted in a rapid decrease in YAP and TAZ protein levels, which reached less than 50% of the normal levels after 2 h and less than 30% after 6 h. In contrast, after suppression of *FAT1* expression, basal YAP and TAZ levels increased and protein synthesis inhibition had a much weaker effect on the cellular YAP/TAZ protein levels (Fig. 5f). These data suggest that FAT1 controls the basal YAP/TAZ protein degradation rate.

### The intracellular domain of FAT1 interacts with the E3 ligase MIB2 and thereby promotes YAP/TAZ protein degradation

**Fig. 6:**
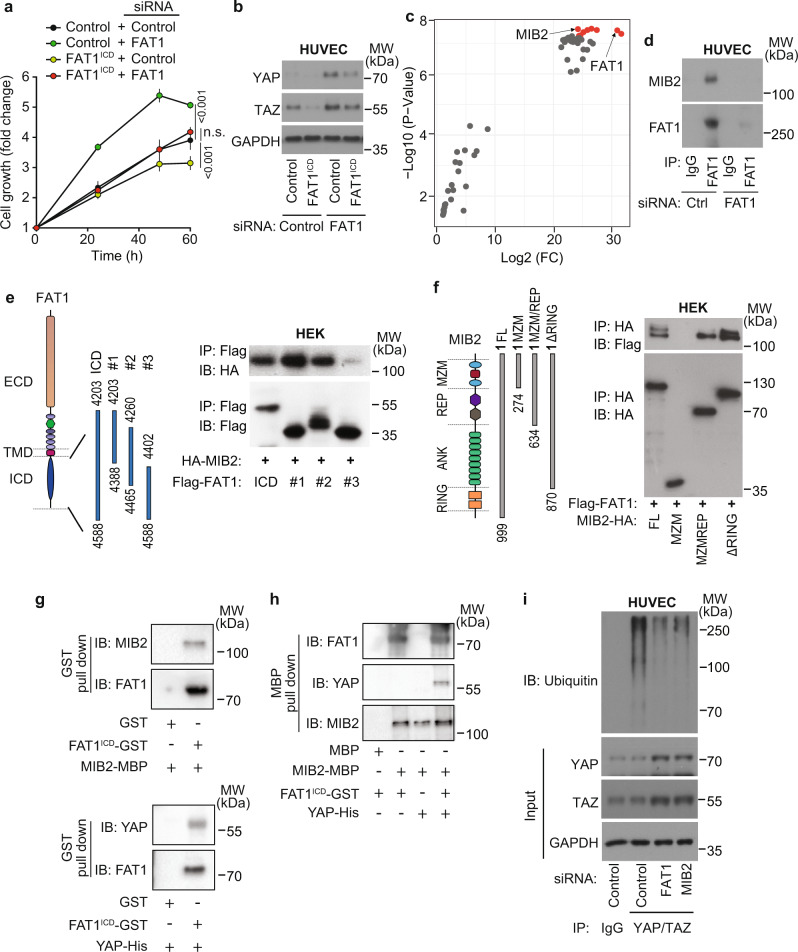
FAT1 interacts with E3 ligases MIB2 to promote YAP/TAZ protein degradation. **a**, **b** HUVECs stably expressing flag-tagged control peptide (control) or flag-tagged intracellular domain of FAT1 fused to the transmembrane domain of the IL-2 receptor (FAT1^ICD^) were transfected with control siRNA or siRNA directed against endogenous *FAT1*, and cell proliferation (**a**) or YAP and TAZ protein levels (**b**) were determined (mean values ± SEM, *n* = 10, two-way ANOVA and Bonferroni’s post hoc test (**a**); 1 representative of 3 independently performed experiments (**b**)). **c** Proteins immunoprecipitated with an anti-FLAG antibody from lysates of HUVECs expressing Flag-tagged FAT1^ICD^ or control were analyzed by mass spectrometry. Shown is a volcano plot highlighting enriched proteins in the FAT1^ICD^ interactome. Statistical evaluation was two-sided Bayesian moderated *t*-test provided by the limma package. The *p* values were adjusted for multiple hypothesis testing using the method by Benjamini–Hochberg. **d** FAT1 was immunoprecipitated from HUVECs transfected with control siRNA or siRNA directed against *FAT1*, and immunoprecipitates were analyzed for presence of MIB2 (IgG: control immunoprecipitation; shown is a representative of three independently performed experiments). **e**, **f** HEK293 cells were cotransfected with HA-tagged MIB2 and the indicated Flag-tagged FAT1-ICD mutants (**e**) or with Flag-tagged FAT1 and the indicated HA-tagged mutants of MIB2 (**f**). Thereafter FAT1 (**e**) or MIB2 (**f**) was immunoprecipitated (IP) using anti-Flag or anti-HA antibodies, respectively, and precipitates were analyzed with the indicated antibodies (IB). Shown are a representative of three independently performed experiments. **g**, **h** The purified FAT1^ICD^-GST fusion protein was incubated with purified MBP-tagged MIB2 or His-tagged YAP protein followed by GST pull-down (**g**) or purified MBP-MIB2 fusion protein was incubated with purified FAT1^ICD^-GST and His-tagged YAP protein followed by MBP pull-down (**h**). Precipitated proteins were analyzed by immunoblotting (IB) using the indicated antibodies. Shown are a representative of three independently performed experiments. **i** HUVECs were transfected with control siRNA or siRNA directed against *FAT1* or *MIB2*, and YAP/TAZ were immunoprecipitated (IP). Unrelated IgG served as an IP-control. Ubiquitination of YAP/TAZ was analyzed by immunoblotting (IB) using an anti-ubiquitin antibody. Immunoblots of the lysates (input) using the indicated antibodies are shown below (1 representative of 3 independently performed experiments).

In order to understand how FAT1 increases YAP/TAZ protein degradation, we tested whether the intracellular domain of FAT1 was sufficient to rescue the effect of FAT1 knock-down in endothelial cells in vitro. As shown in Fig. 6a, b, the increased growth of HUVECs as well as the increase in YAP and TAZ protein levels induced by FAT1 knock-down were rescued by re-expression of the FLAG-tagged intracellular domain of FAT1 fused to the transmembrane domain of the IL-2 receptor (FLAG-IL-2R-FAT1-ICD). Next, we searched for potential interaction partners of the FAT1 intracellular domain involved in regulating YAP/TAZ protein turnover. To this end, we expressed FLAG-IL-2R-FAT1-ICD in HUVECs to perform a mass spectrometry analysis following FLAG immunoprecipitation. This analysis revealed several highly enriched proteins in the anti-FLAG-IL-2R-FAT1-ICD precipitates including the E3 ligase MIB2 (Fig. 6c and Supplementary Table 1). We confirmed that MIB2 coprecipitates with endogenous wild-type FAT1 in HUVECs (Fig. 6d). We then tested the ability of different FAT1 mutants to interact with MIB2. These mutants lack parts of the intracellular portion of FAT1, which ranges from amino acids 4203–4588. We found that fragments 4203–4388 and 4260–4465 but not fragment 4402–4588 interacted with MIB2, indicating that MIB2 interacts with a region of the intracellular part of FAT1, which is located between amino acids 4260 and 4402 (Fig. 6e). When HA-tagged MIB2 was co-expressed with FLAG-tagged FAT1 in HEK293 cells, precipitation of FAT1 led to a specific pull-down of MIB2 (Fig. 6f). Coexpression of FAT1 with different mutants of MIB2 and subsequent co-immunoprecipitation experiments showed that the part of MIB2 containing MZM and REP domains was sufficient to precipitate FAT1 while the MZM domain alone was unable to do so. These data suggest that the interaction requires the REP domain of MIB2 (Fig. 6f). In pull-down assays using purified MIB2 and YAP as well as the intracellular domain of FAT1 (FAT1^ICD^) fused to GST, we found that FAT1^ICD^ specifically interacted with MIB2 and YAP (Fig. 6g). Also purified MIB2 fused to MBP was able to interact with the intracellular part of FAT1, whereas it was only able to stably interact with His-tagged YAP in the presence of the intracellular part of FAT1 (Fig. 6h). To test whether the interaction of MIB2 and FAT1 affected the ubiquitination of YAP/TAZ, we immunoprecipitated YAP and TAZ from HUVECs and monitored their ubiquitination. We saw that the level of ubiquitination found in control cells was strongly reduced after suppression of *FAT1* or *MIB2* expression using specific siRNAs (Fig. 6i and Supplementary Fig. 2a, b). Taken together, these data indicate that FAT1 interacts with MIB2 to promote YAP/TAZ ubiquitination.

### MIB2 mediates FAT1-dependent YAP/TAZ protein degradation

**Fig. 7:**
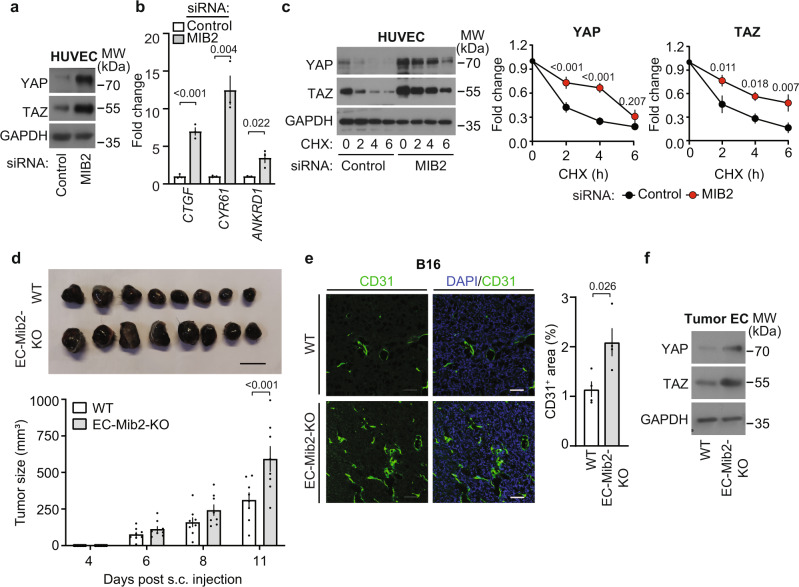
Role of MIB2 in endothelial cells in vivo and in vitro. **a**–**c** HUVECs were transfected with control siRNA or siRNA directed against *MIB2* as indicated. 24 h later, YAP/TAZ protein levels were analyzed by immunoblotting (**a**), YAP/TAZ target genes were analyzed by qRT-PCR (**b**) and YAP and TAZ protein degradation was determined after incubation of cells with 50 µg/ml cycloheximide (CHX) for the indicated time periods and subsequent immunoblotting using anti-YAP/TAZ antibodies (**c**) (*n* = 3 independent experiments in **b** and **c**). **d** Tumor development after subcutaneous injection of B16 melanoma cells into control and EC-Mib2-KO mice (*n* = 8 mice per group). Bar length: 1 cm. **e** Vascularization of LLC1 tumors was determined after staining with DAPI and an anti-CD31 antibody. The bar diagram shows the statistical analysis (*n* = 4 mice per group; 10–15 randomly selected sections were analyzed per mouse). Scale bar: 50 µm. **f** YAP and TAZ protein levels in endothelial cells isolated from primary LLC1 tumors of control and EC-Mib2-KO mice. Shown is a representative immunoblot of three independent experiments. Shown are mean values ± SEM. (two-tailed unpaired *t*-test (**b**, **e**) or two-way ANOVA and Bonferroni’s post hoc test (**c**, **d**)).

We then tested the effect of an siRNA-mediated knock-down of MIB2 on YAP/TAZ levels and activity in HUVECs. Knock-down of the E3-ligase resulted in an increase in YAP and TAZ protein levels (Fig. 7a). This was accompanied by an increased expression of YAP/TAZ target genes (Fig. 7b), similar to the effects of FAT1 suppression. Furthermore, knock-down of MIB2 also prevented the rapid decrease in YAP and TAZ protein levels after inhibition of protein synthesis by cycloheximide in HUVECs (Fig. 7c). To analyze the consequences of an endothelial loss of MIB2 under in vivo conditions, we generated tamoxifen-inducible endothelium-specific MIB2-deficient mice (*Tek-CreERT2;Mib2*^*flox/flox*^, herein referred to as EC-Mib2-KO). When testing the effect of endothelial MIB2 deficiency on B16 melanoma tumor growth, we observed a significantly increased tumor volume after subcutaneous injection of tumor cells in EC-Mib2-KO mice (Fig. 7d). Histological analysis of the tumors revealed an increased vessel area in tumors of EC-Mib2-KO mice (Fig. 7e). This was accompanied by increased YAP and TAZ protein levels in tumor endothelial cells isolated from EC-Mib2-KO mice compared to tumor endothelial cells from control animals (Fig. 7f). Thus, EC-Mib2-KO mice recapitulate the phenotype of EC-Fat1-KO animals.

**Fig. 8:**
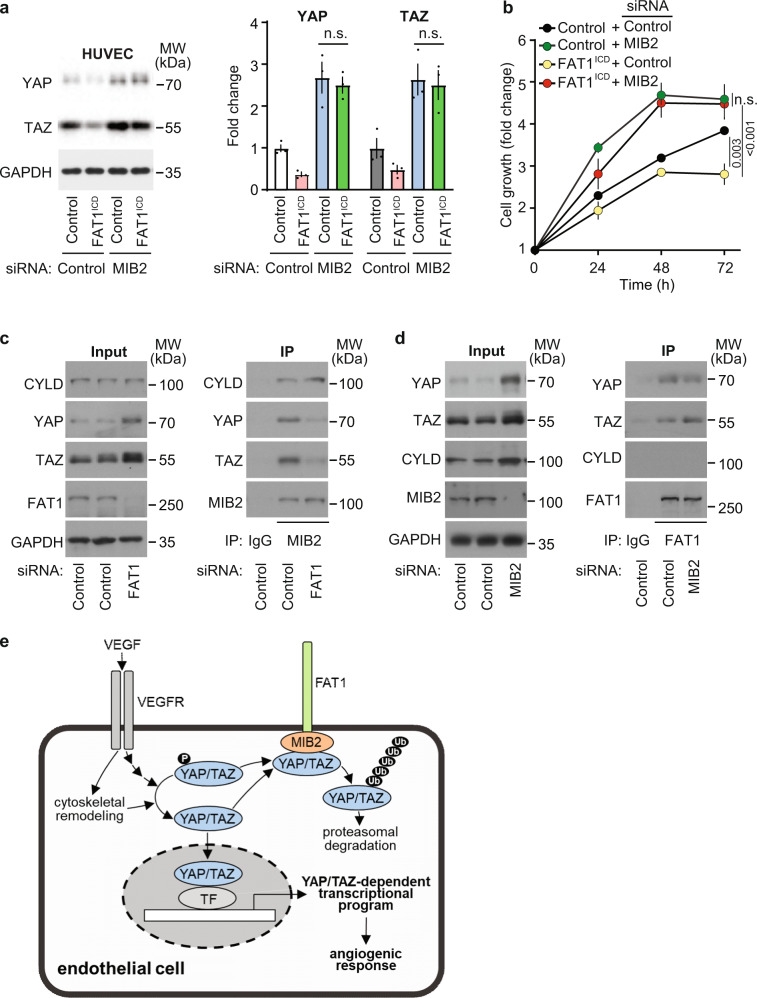
MIB2 mediates FAT1-dependent YAP/TAZ regulation. **a**, **b** Control and FAT1^ICD^-expressing HUVECs were transfected with control siRNA or siRNA directed against *MIB2*, and YAP and TAZ protein levels (**a**) or cell proliferation (**b**) were determined by immunoblotting or a colorimetric cell proliferation assay, respectively (*n* = 3 (**a**) and *n* = 4 (**b**) independently performed experiments). **c**, **d** HUVECs were transfected with control siRNA or siRNA directed against *FAT1* (**c**) or *MIB2* (**d**), and cells were lyzed to either detect the indicated proteins by Western blotting (input) or lysates were subjected to immunoprecipitation (IP). Immunoprecipitation was performed using an anti-MIB2 (**c**) or an anti-FAT1 antibody (**d**). Subtype-matched IgG served as control. Immunoprecipitates were analyzed by immunoblotting using the indicated antibodies. Shown is a representative of three independently performed experiments. **e** Schematic representation showing how FAT1 negatively controls endothelial cell proliferation during angiogenesis through MIB2-mediated degradation of YAP/TAZ. TF transcription factor, VEGF vascular endothelial growth factor, VEGFR VEGF-receptor, Ub ubiquitin. Shown are mean values ± SEM. n.s. not significant (two-tailed unpaired *t*-test (**a**) or two-way ANOVA and Bonferroni’s post hoc test (**b**)).

To test whether MIB2 acts downstream of FAT1 to mediate FAT1-dependent downregulation of YAP and TAZ protein levels, we explored whether the reduction in YAP and TAZ protein levels induced by overexpression of the intracellular domain of FAT1 was affected by knock-down of MIB2. As shown in Fig. 8a, loss of MIB2 expression not only resulted in increased YAP and TAZ protein levels under control conditions but also abolished the effect of FAT1 overexpression on YAP and TAZ protein levels. Similarly, the reduced proliferation of cells overexpressing the intracellular domain of FAT1 was increased after knock-down of MIB2 and reached levels similar to those seen after knock-down of MIB2 in control cells (Fig. 8b). We then tested whether the protein level of another substrate of MIB2 was also affected by loss of FAT1 and found that knock-down of FAT1, while increasing protein levels of YAP and TAZ, had no effect on protein levels of CYLD, a known substrate of MIB2^39^. In contrast, knock-down of MIB2 increased protein levels of all 3 proteins (Fig. 8c, d). This indicates that MIB2 requires FAT1 to increase degradation of YAP and TAZ, but not of CYLD. This is also supported by immunoprecipitation experiments, which showed that YAP and TAZ, but not CYLD, were co-precipitated with FAT1, whereas all 3 proteins were co-precipitated with MIB2 (Fig. 8c, d and Supplementary Fig. 2c). Co-precipitation of YAP and TAZ by MIB2 was strongly reduced after knock-down of FAT1. However, co-precipitation of CYLD and MIB2 was not affected by suppression of *FAT1* expression (Fig. 8c). Interestingly, co-precipitation of YAP and TAZ by FAT1 was not affected by knock-down of MIB2 (Fig. 8d). This indicates that YAP and TAZ require FAT1 to serve as substrates for MIB2, which also interact with FAT1 (Fig. 8e).

## Discussion

Several lines of evidence have indicated that FAT1 is a negative regulator of YAP/TAZ. Inactivating mutations in the tumor suppressor gene *FAT1* have been shown to promote tumor growth and to increase YAP/TAZ signaling^10,11^. Similarly, loss of Fat1 in the zebrafish pronephros leads to cyst formation and increased YAP signaling^15^, and decreased neuronal differentiation after loss of FAT1 is accompanied by increased YAP activity^14^. How FAT1 regulates YAP/TAZ has remained unclear. Recent evidence has indicated that FAT1 expressed by tumor cells can activate the Hippo pathway by serving as a scaffold to assemble several components of the Hippo signaling pathway^10^ and that FAT1 inhibits CAMK2-mediated nuclear translocation of YAP/TAZ through SRC/YES kinases^13^. Our data show that FAT1 in endothelial cells regulates YAP and TAZ in a Hippo kinase-independent manner. Endothelial FAT1 promotes YAP/TAZ protein degradation by interaction with the E3 ligase MIB2, which stimulates degradation of YAP/TAZ protein, and loss of this mechanism leads to increased YAP/TAZ protein levels, YAP/TAZ target gene expression as well as to increased endothelial proliferation (Fig. 8e).

Several mechanisms of ubiquitination and subsequent proteasomal degradation of YAP and TAZ have been described. The Hippo pathway effector kinase LATS1/2 phosphorylates YAP and TAZ at serine 381 and 311, respectively, thereby priming the proteins for further phosphorylation by casein kinase 1 (CKδ/ε). The phosphorylated YAP and TAZ then recruit the β-TrCP subunit of the SCF^β-TrCP^ E3 ubiquitin ligase, leading to their ubiquitination and degradation^40,41^. TAZ, but not YAP, can recruit β-TrCP also at an N-terminal site in a Hippo pathway-independent manner after phosphorylation by GSK3 kinase, which is inhibited by phosphoinositide-3-kinase (PI-3-K), thereby leading to TAZ ubiquitination and degradation^42^. This GSK3-kinase-mediated degradation is believed to mediate stabilization of TAZ protein levels in tumors with high phosphoinosite-3-kinase (PI-3-K) signaling activity or in PTEN mutant cancer cells^42^. In addition to β-TrCP-dependent ubiquitination, human YAP has also been shown to be ubiquitinated by the E3 ligase F-box and WD repeat domain-containing 7 (Fbxw7) in hepatocellular carcinoma cells, in which Fbxw7 levels correlate inversely with YAP protein levels^43^. Similarly, in breast cancer cells, the E3 ligase RNF187 leads to ubiquitination of YAP, and RNF187 levels correlate inversely with YAP protein levels^44^. Besides these ubiquitination processes resulting in proteasomal degradation of YAP/TAZ, YAP has also been shown to be subject to non-proteolytic ubiquitination by the SCF^SKP2^ E3 ligase complex (SKP2) which promotes nuclear localization and activity^45^. Our data extend the repertoire of E3 ubiquitin ligases controlling YAP/TAZ protein turnover and show that MIB2 governs YAP/TAZ protein levels downstream of the essential protocadherin FAT1.

MIB2 is an E3 ligase, which has recently been shown to ubiquitinate RIPK1, resulting in the inhibition of the pro-apoptotic activity of RIPK1 and the inhibition of cell death triggered by various stimuli including TNFα^46^. MIB2 has also been shown to increase TNFα-induced NF-κB signaling by mediating ubiquitination of the deubiquitination enzyme CYLD, resulting in its proteasomal degradation^39^. To what degree MIB2-mediated effects on RIPK1 or CYLD signaling contribute to the effects of FAT1 is unknown. An involvement to CYLD in FAT1-dependent regulation of YAP and TAZ appears unlikely, since CYLD downregulation rather correlates with enhanced nuclear localization of YAP/TAZ and increased YAP/TAZ target gene expression^47^. This is supported by our data showing that suppression of *FAT1* expression had no effect on CYLD protein levels. CYLD, in contrast to YAP/TAZ, was also not co-precipitated with an anti-FAT1-antibody. This indicates that FAT1 interacts with YAP/TAZ but not with CYLD. We also showed that the purified intracellular part of FAT1 can directly interact with YAP and MIB2, and that the interaction between FAT1 and YAP/TAZ was not affected by knock-down of MIB2. Since interactions between E3 ligases and their substrates are known to be weak and very transient^48–50^, we were not able to co-precipitate MIB2 and YAP directly. These data suggest that FAT1 interacts with both MIB2 and YAP/TAZ and that the functional interaction of MIB2 with YAP/TAZ requires FAT1 (Fig. 8e).

YAP and TAZ have been shown to be required for developmental as well as for tumor angiogenesis^24–29,51^. Nuclear translocation and activation of endothelial YAP/TAZ during angiogenesis can be induced by VEGF, VE-cadherin, adherens junctions, cytoskeletal remodeling or lysophosphatidic acid LPA_4_/LPA_6_ receptor-mediated Rho/Rho-kinase signaling^25,27,28,30,34,52^. VEGF signaling stimulates YAP/TAZ through cytoskeletal remodeling and inactivation of the Hippo effectors LATS1/2^25,53^. While these studies have focused on the analysis of YAP/TAZ activating pathways, our data identify a major YAP/TAZ inactivating mechanism involving FAT1/MIB2-mediated YAP/TAZ protein degradation. Whether FAT1/MIB2-mediated YAP/TAZ regulation is itself controlled is currently not clear. However, our analysis of FAT1 levels during endothelial cell culture suggests that FAT1 protein levels can in turn be regulated, resulting in changes in YAP/TAZ protein turnover and activity.

Endothelium-specific loss of YAP/TAZ has been shown to result in impaired tumor and developmental angiogenesis^24–26,29,51^, whereas endothelium-specific overexpression of YAP or TAZ increased angiogenesis^28,29,54^. Consistent with a general role of FAT1 in the negative regulation of endothelial YAP/TAZ, we found that loss of FAT1 resulting in YAP/TAZ activation increased endothelial cell proliferation during postnatal retinal angiogenesis as well as in tumor angiogenesis. Despite continuously elevated YAP/TAZ protein levels in the absence of FAT1, endothelial cells did not show completely unrestricted proliferation, indicating that additional mechanisms are involved in suppressing endothelial cell proliferation. Similar to endothelium-specific YAP overexpression^28^, increased YAP/TAZ protein levels due to loss of FAT1 had no effect on the vasculature of quiescent tissue in adult animals.

In addition to developmental and tumor angiogenesis, we found that loss of FAT1 also affected post-ischemia neovascularization. After both myocardial infarction and hind limb ischemia, EC-Fat1-KO mice showed increased endothelial cell proliferation. This is consistent with a recent report showing that endothelial loss of YAP resulted in impaired angiogenesis after myocardial infarction^55^. However, while increased proliferation of tumor endothelial cells in EC-Fat1-KO mice resulted in increased tumor growth, indicating improved angiogenesis and tissue perfusion, increased angiogenesis after myocardial infarction did not seem to promote tissue perfusion and rather led to an impaired recovery. This indicates that upregulation of endothelial YAP/TAZ activity and increased endothelial cell proliferation does not necessarily improve angiogenesis and subsequent tissue perfusion but that additional factors influence the relationship between endothelial cell proliferation and vascular network formation.

Our data identify a novel mechanism of YAP/TAZ activity regulation in endothelial cells, in which the protocadherin FAT1 controls YAP/TAZ protein degradation by interacting with the E3 ligase MIB2. FAT1 thereby promotes YAP/TAZ degradation and negatively controls endothelial proliferation, a mechanism which could serve as a target for therapies aiming at inhibiting angiogenesis.

## Methods

### Reagents

Tamoxifen (catalog #T5648), 4-hydroxy tamoxifen (catalog #H7904), MG132 (catalog #M8699), N-ethylmaleimide (NEM) (catalog #E3876), cycloheximide (catalog #239763), reduced L-glutathione (catalog #G4251) and D-maltose (catalog #M9171) were purchased from Sigma-Aldrich. Antibodies directed against ERG (catalog #ab92513), CD31 (catalog #ab7388), FAT1 (catalog #ab109202), FAT4 (catalog #ab130076) were from Abcam. Isolectin GS-IB4 (catalog #I21413/I32450) was from Life Technologies GmbH. Antibodies directed against HA (catalog #H6533), Flag M2 (catalog #A8592) were from Sigma-Aldrich. Antibodies directed against YAP/TAZ (catalog #8418), YAP (catalog #4912), Lamin A/C (catalog #2032), GAPDH (catalog #2118), LATS1 (catalog #3477), phospho-LATS1 (S909) (catalog #9157), phospho-LATS1 (catalog #1079) (catalog #8654), phospho-MST1 (T183) (catalog #3681) phospho-MOB1 (T35) (catalog #8699) Ubiquitin (catalog #3936S), Cleaved Caspase-3 (catalog #9664), CYLD (catalog #8462) were obtained from Cell Signaling. The MIB2 antibody (catalog #A301-414A) was from Bethyl Laboratories, and Matrigel (catalog #356231) was purchased from Merck. Isopropyl-β-D-1-thiogalactopyranosid (IPTG) (catalog #CN08.2) and imidazole (catalog #X998.3) were from Carl Roth GmbH.

### Plasmids

Plasmid pCDH-EF1a-GaussiaSP-MCS-IRES-copGFP carrying a cDNA encoding the extracellular and transmembrane domains (amino acids 1–259) of the human interleukin-2 receptor fused with the intracellular domain of FAT1 (amino acid 4203–4588) and carrying a FLAG tag at the C-terminus (IL2R-FAT1-ID-Flag) and an empty plasmid were purchased from VectorBuilder.

To generate different fragments of the intracellular part of FAT1, the respective sequences encoding amino acids 4203–4588, 4203–4388, 4260–4465 and 4402–4588 were amplified from the IL2R-FAT1-ID-Flag cDNA using primers carrying EcoRV/XhoI restriction sites and subcloned into the EcoRV/XhoI site of the vector pcDNA3 (Invitrogen). The pcDNA3 eukaryotic expression plasmids carrying the cDNA encoding different fragments of MIB2^46^ were a gift from Pascal Meier (The Institute of Cancer Research, London, UK).

### Cell culture

HUVECs were purchased from Gibco. HUAECs were from Provitro. HUVECs and HUAECs were cultured in EGM-2 medium (CC-3156, Lonza) containing supplements (CC-4176 or CC-4147), respectively, and were used for a maximal number of 6 passages. All cells were cultured at 37 °C and 5% CO_2_ and were tested negative for mycoplasma contamination before experiments.

### 3D-cell culture

The pre-thawed matrigel was applied to a μ-Slide Angiogenesis well (Ibidi, Catalog #81506) according to the manufacturer’s instructions. HUVECs transfected with the indicated siRNA were then seeded onto the matrigel, and pictures were captured by a motorized inverted microscope (Olympus IX81).

### siRNA-mediated knockdown

Cells at 50–70% confluence were transfected with siRNA using Opti-MEM and Lipofectamine RNAiMAX (Invitrogen) as described previously^56^. Control siRNA and siRNAs directed against *FAT1*, *FAT4*, *MIB2*, *YAP*, and *TAZ* were from Sigma. The targeted sequences of those siRNAs were as follows:

*FAT1* (SASI_Hs01_00232012), 5-CATCGAACAGGCCAATGAA-3; *FAT4* (SASI_Hs02_00356912), 5-CTAACAACCACGGAACTTT-3; *MIB2* (SASI_Hs01_00031060), 5-GTGTGTGCCTGGACTACGA-3; *YAP* (SASI_Hs01_00182402), 5-CACCTATCACTCTCGAGAT-3; *TAZ* (SASI_Hs01_00124477), 5-GATGAATCAGCCTCTGAAT-3.

### Western blotting

Cells were directly lysed in 1 × Laemmli buffer containing 150 mM NaCl, 50 mM Tris-HCl (pH 6.8), 2.5 mM EDTA, 1% (w/v) SDS, 5% glycerol, 1% β-mercaptoethanol, 5% (w/v) bromophenol blue. Total cell lysates were boiled for 10 min and were subjected to sodium dodecyl sulfate-polyacrylamide gel electrophoresis (SDS-PAGE). Protein was then transferred to nitrocellulose membranes, followed by overnight incubation with primary antibodies. Membranes were incubated with horseradish peroxidase-conjugated (HPC) conjugated secondary antibodies directed against rabbit or mouse IgG (Cell Signaling Technology, catalog #7074 and #7076, 1:3000) for 2 h at room temperature and were developed using the ECL detection system (Thermo Scientific Pierce, Life Technologies). Protein band intensities were analyzed by ImageJ software (NIH). In some cases, cells were fractionated to distinguish nuclear and cytoplasmic protein content prior to Western blot analysis. Cell fractionation was performed using the NE-PER extraction reagent (Thermo Fisher Scientific, catalog #78833) according to the manufacturer’s instructions.

### Immunoprecipitation

For immunoprecipitation of endogenous FAT1, HUVECs were lysed in immunoprecipitation (IP) buffer (150 mM NaCl, 25 mM Tris-HCl (pH 7.4), 1 mM EDTA, 1% NP-40, 5% glycerol) supplemented with protease inhibitor cocktail (catalog #4693159001, Roche) on ice. Samples were centrifuged at 30,000 × *g* for 15 min. Supernatant was incubated with 2 µg of the anti-FAT1 antibody overnight at 4 °C on a rotating platform. Thereafter, A/G-Sepharose beads (catalog #sc-2003, Santa Cruz) pre-equilibrated in IP buffer were added and incubated for 1 h at 4 °C. A/G-Sepharose beads were then collected by centrifugation at 3500 × *g* for 1 min, and proteins were eluted with Laemmli buffer after being washed three times. Samples were subjected to SDS-PAGE using VeriBlot IP detection reagent (catalog #ab131366, abcam). For immunoprecipitation of heterologously expressed proteins, HEK-293 cells were transfected with eukaryotic expression plasmids carrying the indicated cDNAs for 24–36 h using Lipofectamine 2000 (catalog #11668019, Thermo Fisher Scientific) and were then processed as described above.

### RNA isolation and quantitative RT-PCR

Total RNA was isolated from cells using Quick-RNA ^TM^ MicroPrep Kit (Zymo Research). Quality control of RNAs were done with Nanodrop ND-100 Spectrophotometer. RNA was reverse-transcribed using ProtoScript II First Strand cDNA Synthesis Kit (New England Biolabs) according to manufacturer’s instructions. Quantitative RT-PCR was done with TaqMan Probe and primers were designed with the Roche online tool and a Universal Probe Library assay (Roche). Relative expression levels were calculated after normalizing with GAPDH expression levels. Copy numbers were calculated as described^56^. The following primer sequences were used: *FAT1* (human) #81: 5’-GAGCTGCCGAGGACTTTAGA-3’, 5’-TGCTTAACTGTCGGGAATCA-3’; *FAT4* (human) #48: 5’- GTCATGGCAGCAGTGTCATT-3’, 5’-AGGCAGTGTCAGGAGTAACAGAT-3’; *MIB2* (human) #11: 5’-TACAAGGACCACCTCCCAAG-3’, 5’-TGTCCAGCAGACACTTGACC-3’; *CYR61* (human) #7: 5’-TCCAGGGCACACCTAGACA-3’, 5’-GCCCATTTTCTCCATGATTC-3’; *ANKRD1* (human) #61: 5’-GATCGAATTCCGTGATATGCT-3’, 5’-AAACATCCAGGTTTCCTCCA-3’; *CTGF* (human) #85: 5’-GCCTCCTGCAGGCTAGAGA-3’, 5’-GATGCACTTTTTGCCCTTCT-3’; *YAP* (human) #47: 5’-ATCCCAGCACAGCAAATTCT-3’, 5’-TGGATTTTGAGTCCCACCAT-3’; *TAZ* (human) #7: 5’-ATTCGAATGCGCCAAGAG-3’, 5’-AACTGGGGCAAGAGTCTCAG-3’; *GAPDH* (human) #82: 5’-GCATCCTGGGCTACACTGA-3’, 5’-CCAGCGTCAAAGGTGGAG −3’. *Fat1* (mouse) #46: 5’-CATCAATGATAACCCTCCTGTG-3’, 5’-GCTTCAGACAGGGTTGTGG-3’; *Fat2* (mouse) #47: 5’-CATTCAGGTGACTGCCAATG-3’, 5’-AGCCTGTAGCTCACCTGTCC-3’; *Fat3* (mouse) #72: 5’-CTGCTGTGCAAGTTGATGCT-3’, 5’-TTTGTCGATGGCTGTTACCC-3’; *Fat4* (mouse) #32: 5’-CAGGACCCGGATGTGTTAGA-3’, 5’-GAGGCTGGTGACTCCTGAAG-3’.

### Determination of cell growth and proliferation

To evaluate cell growth, we used a colorimetric cell proliferation assay (CellTiter 96® AQueous One Solution Cell Proliferation Assay; Catalog #G3580, Promega). In total, 4000–8000 HUVECs or HUAECs were seeded on 96-well plates and siRNA-mediated knock-down was performed once cells were attached. Six hours later, cell proliferation was determined according to the manufacturer’s instructions. Absorbance was measured at 490 nm using a Synergy 2 microplate reader (BioTek). Alternatively, cell proliferation was determined using the IncuCyte®ZOOM life cell imaging system (Essen Bioscience, Ann Arbor, MI, USA). Cells were seeded on 48- or 96-well plates, and siRNA-mediated knock-down was performed on the following day. Medium was changed 6 h after transfection and time was set as 0 h. Cell growth was monitored for the indicated time periods. Growth rates were calculated as a percentage of cell confluence per image and over time.

### Cycloheximide chase experiment

HUVECs were transfected with the indicated siRNAs following the protocol mentioned above. In total, 48 h after the first transfection, cells were treated with 50 µg/ml cycloheximide for the indicated time periods and then collected in 1 × Laemmli blue buffer directly for further analysis by immunoblotting.

### Determination of ubiquitination

HUVECs were transfected with siRNAs following the protocol described above and after 12 h were treated with 20 µM MG132 for another 12 h. Cells were then lysed in 100 µl lysis buffer containing 150 mM NaCl, 10 mM Tris-HCl (pH 8.0), 2% SDS, 2 mM sodium orthovanadate, 50 mM sodium fluoride, 20 nM NEM as well as protease inhibitors according to manufacturer’s instructions for 30 min on ice. Whole cell lysates were boiled for 10 min, and 900 µl of dilution buffer (150 mM NaCl, 10 mM Tris-HCl (pH 8.0), 2 mM EDTA, 1% Triton X-100) was added. Samples were centrifuged at 16,000 × *g* for 15 min, and the supernatants were incubated with YAP/TAZ antibody and protein-A- or G-agarose for 4–5 h at 4 °C. Beads were spun down at 500 × *g* for 5 min and were washed twice with dilution buffer. Samples were then dissolved in 1 × Laemmli buffer, and ubiquitination was analyzed by Western blotting using an anti-ubiquitin antibody.

### Protein purification from prokaryotic system

Bacterial protein expression vectors (pET) carrying the cDNA encoding the FAT1 intracellular domain (amino acids 4203–4588) fused with glutathion-S-transferase (GST), MIB2 fused to maltose binding protein (MBP) or His-tagged YAP were obtained from VectorBuilder, and Rosetta DE3 component bacteria were transformed with the plasmids. The transformed bacteria were initially cultured in 100 ml LB overnight and were then transferred to 1 l LB and cultured until an OD (600 nm) of 0.8 was reached. Protein expression was induced by addition of IPTG to a final concentration of 1 mM, and the bacteria were incubated at 16 °C overnight. Cells were harvested by centrifugation at 4500 × *g* at 4 °C for 5 min. Bacterial pellets were resuspended in 10 ml ice-cold lysis buffer (for His-tagged YAP and GST-tagged FAT1^ICD^: 50 mM Tris-HCl (pH 7.5), 150 mM NaCl, 5 mM MgCl_2_ and 1 mM DTT; for MBP-tagged MIB2: 20 mM Tris-HCl (pH7.4), 200 mM NaCl, 1 mM EDTA and 1 mM DTT) and the suspension was sonicated with an ultrasonic homogenizer (BANDELIN, UW2070) at 50% amplitude for 4 × 25 s at 4 °C. Lysates were incubated for 30 min at 4 °C under constant agitation using an over-head shaker and were then centrifuged at 8000 × *g* for 10 min, and supernatants were collected. Washed beads or resins for the pull-down of His-, GST- and MBP-tagged YAP, FAT1^ICD^ and MIB2, respectively, (HisPur^TM^ cobalt resin (catalog #89964, Thermo Fisher Scientific); glutathione agarose (catalog #745500.10, Macherey-Nagel); MBP-binding amylose beads (catalog #E8035, New England Biolabs)) were added at a volume of 1 ml. After incubation for 4–5 h at 4 °C under constant agitation using a rotator (VWR), the beads were washed three times with lysis buffer, and proteins were then eluted with individual elution buffers (for His-tagged YAP: 50 mM Tris-HCl (pH8), 500 mM NaCl and 500 mM Imidazol; for GST-tagged FAT1^ICD^: 125 mM Tris-HCl (pH7.4), 150 mM NaCl, 0.1% (v/v) Triton X-100, 50 mM reduced glutathione and 1 mM DTT; for MBP-tagged MIB2: 20 mM Tris-HCl (pH7.4), 200 mM NaCl, 1 mM EDTA, 1 mM DTT and 10 mM maltose). The concentration of the purified proteins was determined by measuring the absorbance at 280 nm and by SDS-PAGE after two times dialysis against 2 l of PBS in 4 °C.

### GST/MBP pull-down assay

A mixture of the bait proteins (GST or MBP-fused protein) and prey proteins were incubated in PBS at a concentration of 10 µM each in a volume of 100 µl at room temperature for 1 h and for an additional period of 2 h on a rotator at 4 °C after addition of 300 µl pre-washed GST or MBP binding beads in PBS. After centrifugation at 500 × *g* for 5 min, supernatants were removed, and beads were washed 2 times with PBS. Thereafter, proteins were eluted with PBS containing 10 mM glutathione (for GST fused proteins) or 10 mM maltose (for MBP tagged proteins). The eluted proteins were then analyzed by Western blotting using anti-bait protein and anti-prey protein antibodies. As a negative control, parallel experiments were performed using GST and MBP alone together with prey proteins.

### Proteomics analysis

Samples for proteomics were obtained from HUVECs transduced with the FAT1 intracellular part fused with the extracellular part and transmembrane domain of the IL-2 receptor and carrying a Flag tag on the C terminus (see above) for 36 h, followed by lysis in IP buffer. Lysates were incubated with magnetic anti-FLAG beads (M8823, Sigma/Aldrich) overnight at 4 °C. Affinity purified samples were subjected to in-gel (4–12% NuPAGE Bis-Tris gels, Thermo Fisher Scientific) digestion as described^57^. In brief, gel lanes were cut into nine blocks/fractions and finely diced. Embedded proteins were reduced (10 mM dithiothreitol) and alkylated (55 mM iodoacetamide), followed by overnight digestion using trypsin (Serva). Peptides were extracted by increasing concentrations of acetonitrile and lyophilized. Final desalting, concentration and storage utilized “stop and go extraction” (STAGE) tips^58^. Eluted peptides where subjected to electrospray ionization (ESI)-mediated liquid chromatography/tandem mass spectrometry (LC/MS2) using in house-packed column emitters (15 cm length, 70 µm ID, 1.9 µm ReprsoSil-Pur 120 C18-AQ, Dr. Maisch) and a buffer system comprising solvent A (0.1% formic acid) and solvent B (80% acetonitrile, 0.1% formic acid). Instrumentation details and parameters were extracted and summarized using MARMoSET^59^ and are repository deposited (see below). Peptide/spectrum matching and label free quantitation were performed using the MaxQuant suite of algorithms^60–62^ against the human UniProt database (canonical & isoforms; downloaded on 2019/01/23). Parameters are included in the repository deposition (ProteomeXchange Consortium (http://proteomecentral.proteomexchange.org) via the MASSIVE partner repository [10.1093/nar/gkz984] with the dataset identifier PXD040114). Downstream statistical analysis used the limma-based in-house package autonomics (10.18129/B9.bioc.autonomics)^63^.

### Animal models

All mice were backcrossed onto a C57BL/6 background at least 8–10 times. Male and female animals (8–20 weeks of age) were both used. Mice were housed under a 12-h light-dark cycle with free access to food and water and under specific pathogen-free conditions unless stated otherwise. Mice carrying a floxed allele of *Fat1* have been described previously^5^. Generation of mice with floxed alleles of *Yap* and *Wwtr1* (*Taz*) has been described previously^64^. Mice carrying a knock-out first allele of the *Mib2* gene (*Mib2*^*tm1a(EUCOMM)Wtsi*^*/IcsOrl*) were obtained from The European Mouse Mutant Archive. To generate a floxed *Mib2* allele, mice were crossed with ACTB-FLPe mice (The Jackson Laboratory, strain #005703)^65^. Mice were crossed with Tek-CreER^T2^ mice^66^ to obtain animals with inducible endothelium-specific deficiency. If not stated otherwise, mice received 1 mg/day tamoxifen dissolved in corn oil on 5 consecutive days to induce recombination. All animals that served as controls for tamoxifen-induced endothelium-specific knock-outs either lacked Cre or the floxed allele and were treated with tamoxifen in the same way. Animal numbers used for each experiment are provided in the figure legends. Maintenance of the animals and experimental procedures were in agreement with the German animal welfare legislation and were approved by the local animal welfare authorities and committees with the proposal numbers B2-1102 and B2-2005 (Regierungspräsidium Darmstadt, Germany).

### Retinal angiogenesis

For the analysis of angiogenesis in postnatal mouse retina, Cre-mediated recombination was induced in newborn mice by intraperitoneal (i.p.) injections of 50 µl 4-hydroxy-tamoxifen (1 mg/ml) from postnatal day P1 to P3. Eyes were harvested at P6 for further immunostaining analysis. Control animals were littermate animals without Cre expression, which also received 4-hydroxy-tamoxifen.

### Syngeneic tumor models

For primary tumor models, 10^6^ B16 or LLC1 tumor cells in 50 μl PBS were injected subcutaneously into the shaved flank of mice, which had received tamoxifen 14 days before. Tumor sizes were measured 2–3 times per week as described previously^67^.

### Hind-limb ischemia

To evaluate ischemia-induced angiogenesis by immunostaining of the central part of the gastrocnemius (GC) muscle, animals were analyzed after femoral artery ligation (FAL) as described previously^68^. Briefly, mice were narcotized using isoflurane, and FAL was performed on the left hind-limb of the mice by ligating the femoral artery distal to the origin of the deep femoral branch. After 14 days, mice were narcotized (ketamine 180 mg/kg; xylazine 16 mg/kg) and euthanized by transcardial perfusion with 10 ml of PBS, pH 7.4, containing 100 µg adenosine, 1 µg sodium nitroprusside and 0.05% bovine serum albumin, followed by 10 ml of 3% paraformaldehyde. Gastrocnemius muscle from the ligated left hind limb and the non-ligated hind limb were dissected and processed for further analysis by immunostaining.

### Myocardial infarction (MI) and magnetic resonance imaging (MRI)

Myocardial infarction was induced in 13–14-week-old mice by ligation of the left anterior descending artery (LAD) 2 weeks after tamoxifen injection. Mice were anesthetized with isoflurane followed by tracheal intubation. Mice were then treated with buprenorphine (0.1 mg/kg s.c.) twice daily and metamizole in drinking water (200 mg/kg = 0.8 ml/500 ml) for 3 days. LAD ligation was performed using 8-0 suture (Johnson and Johnson) through the lateral thoracotomy at the third intercostal space. Cardiac MRI measurement for each mouse was performed before and after MI 14 days respectively and hearts were collected 14 days after surgery for further histology and immunofluorescence staining. Cardiac MRI measurements are performed on a 7.0T Bruker Pharmascan (Bruker, Ettlingen, Germany), equipped with a 760 mT/m gradient system, using a cryogenically cooled, 4-channel-phased array element 1H receiver-coil (CryoProbe) and a 72 mm room temperature volume resonator for transmission and the IntraGateTM self-gating tool^69^. The parameters for identification of the ECG were adapted for one heart slice and transferred afterwards to the navigator signals of the remaining slices to guarantee in-phase reconstruction of all pictures. Measurements were based on the gradient echo method (repetition time = 6.2 ms; echo time = 1.3 ms; field of view = 2.20 × 2.20 cm; slice thickness = 1.0 mm; matrix = 128 × 128; oversampling = 100). The imaging plane was localized using scout images showing the 2- and 4-chamber view of the heart, followed by acquisition in short axis view, orthogonal on the septum in both scouts. Multiple contiguous short-axis slices consisting of 8 to 10 slices were acquired for complete coverage of the left and right ventricle. Mice were measured under volatile isoflurane (1.5–2.0% in oxygen and air with a flow rate of 1.0 l/min) anesthesia; the body temperature was maintained at 37 °C by a thermostatically regulated water flow system during the entire imaging protocol. MRI data were analyzed using Medis Suite Qmass digital imaging software (Medis, Leiden, Netherlands).

### Immunostaining and immunohistochemistry

For analysis of tissue vascularization, freshly isolated tissues (tumor, heart or gastrocnemius muscle) were fixed in 4% paraformaldehyde (PFA) overnight at 4 °C and put in 10, 20 and 30% sucrose each for 1 day in the stated order. The tissue was cryopreserved in Tissuetek (Sakura Finetek, Staufen, Germany) and was sectioned at a thickness of 10–15 µm. Slides were incubated with 5% BSA and 0.2% Triton X-100 in PBS for 30 min and then incubated with primary antibody CD31 or IB4 conjugated with Alexa Fluor overnight at 4 °C. After being washed with PBS the next day, sections were incubated with the appropriate donkey anti-rat/rabbit IgG highly cross-adsorbed secondary antibodies AlexaFluor^TM^-488- or AlexaFluor^TM^-594 (catalog #A21208, #A21206, #A21207, #A21209, Thermo Fisher Scientific, 1:200) for 2 h (if the primary antibody was not conjugated). Nuclei were stained with DAPI for 15 min afterwards. After being washed with PBS, slices were mounted in Aqua-polymount (Polyscience, Warrington, PA, USA) and were imaged using confocal laser microscopy (Leica SP5). Quantification was performed by taking 5–7 pictures from each slide followed by analysis with ImageJ.

To analyze retina angiogenesis, whole mount immunostaining of retina was performed as descried previously^70^. In brief, whole mouse eyes were fixed in 4% PFA on ice for 3 h. Eyes were dissected and cut into four leaflets after being washed in PBS. Retinae were blocked in blocking buffer (1% BSA, 0.5% Triton, 5% FCS in PBS) for 1 h at room temperature and incubated with the primary antibody at 4 °C overnight in incubating buffer (blocking buffer diluted in PBS, 1:1). After being washed with PBS containing 0.1% Triton X-100, retinae were incubated with Alexa-Fluor-488/594-conjugated secondary antibodies for 2 h at room temperature. To label proliferating cells in retinae, 50 mg/kg of EdU per animal was injected 6 h before they were sacrificed. Retinae were then processed for EdU staining with Molecular Probes™ Click-iT™ EdU Alexa Fluor™ 555 kit (catalog #C10338, Invitrogen) according to manufacturer’s instructions. After EdU staining, retina staining was continued as described above. After staining and washing, retinae were flat-mounted with ProLong Gold Antifade reagent (catalog #P10144, Life Technologies) and were examined by confocal laser microscopy. The endothelial area and the number of endothelial cells were quantified by determining IB4-positive area and ERG-positive cells, respectively. All images were taken between the main retinal artery and vein. All parameters were quantified in a minimum of eight vascularized fields per sample.

For mouse heart infarct size analysis, tissue sections were stained with picrosirius red according to standard protocols as described^71^. Infarct size ratio was determined based on midline length measurement of position at a distance of 2500 µm from the heart apex with ImageJ^72^.

### Cell sorting and flow cytometry analysis

Mouse lung or subcutaneous tumors were minced with dissecting scissors into small pieces and digested in DMEM containing 1.2 units/ml dispase, 2 mg/ml collagenase II and 5 U per ml DNAse I for 15 min at 37 °C while shaking. Cells were filtered through a 70 micron and a 40 micron strainer and were washed with PBS. After centrifugation at 400 × *g* for 5 min, cell pellets were resuspended in Dulbecco’s phosphate-buffered saline (DPBS) containing antibody CD31-FITC (catalog #558738, Becton Dickinson)/CD31-PE (catalog #12-0311-82, eBioscience) and CD45-PE (catalog #12-0451-82, eBioscience)/ CD45-FITC (catalog #553079, Becton Dickinson) and then incubated for 30 min at room temperature. After being washed with PBS and passed through a 35 micron cap filter on FACS tubes, cells were either purified by cell sorting using JSAN-sorter (Bay Bioscience) or were analyzed using a FACS Canto II (Becton Dickinson) (Supplementary Fig. 3). Flow cytometric data were analyzed by FlowJo software (Tree Star Inc).

### Statistics

Statistical analysis was performed using the GraphPad Prism software (9.3.1) from GraphPad Software Inc. (La Jolla, CA, USA). Values are presented as mean ± SEM; *n* represents the number of independent experiments. Statistical analysis between two groups were performed with an unpaired two-tailed Student’s *t* test, while multiple group comparisons were analyzed with one-way ANOVA followed by Tukey’s post hoc test, unless stated otherwise, and comparisons between multiple groups at different time points were performed using two-way ANOVA followed by Bonferroni’s post-hoc test. A *p* value of less than 0.05 was considered to be statistically significant.

### Reporting summary

Further information on research design is available in the Nature Portfolio Reporting Summary linked to this article.

## Supplementary information

Supplementary Information

Reporting Summary

## Data Availability

The mass spectrometric data generated in this study have been deposited in the ProteomeXchange Consortium via the MASSIVE partner repository (10.1093/nar/gkz984) under the accession code PXD040114. Source data are provided with this paper.

## Source data

Source Data
