# Supplementary material for: Endothelial FAT1 inhibits angiogenesis by controlling YAP/TAZ protein degradation via E3 ligase MIB2
Source: Nat Commun. 2023 Apr 8;14:1980. doi: 10.1038/s41467-023-37671-x (PMC10082778; doi:10.1038/s41467-023-37671-x)
Supplement: Supplementary file 1 — Supplementary Information [file 41467_2023_37671_MOESM1_ESM.pdf]

Supplementary Information

"Endothelial FAT1 inhibits angiogenesis by controlling YAP/TAZ protein degradation via E3 ligase MIB2" (Li et al.)

Supplementary Figures

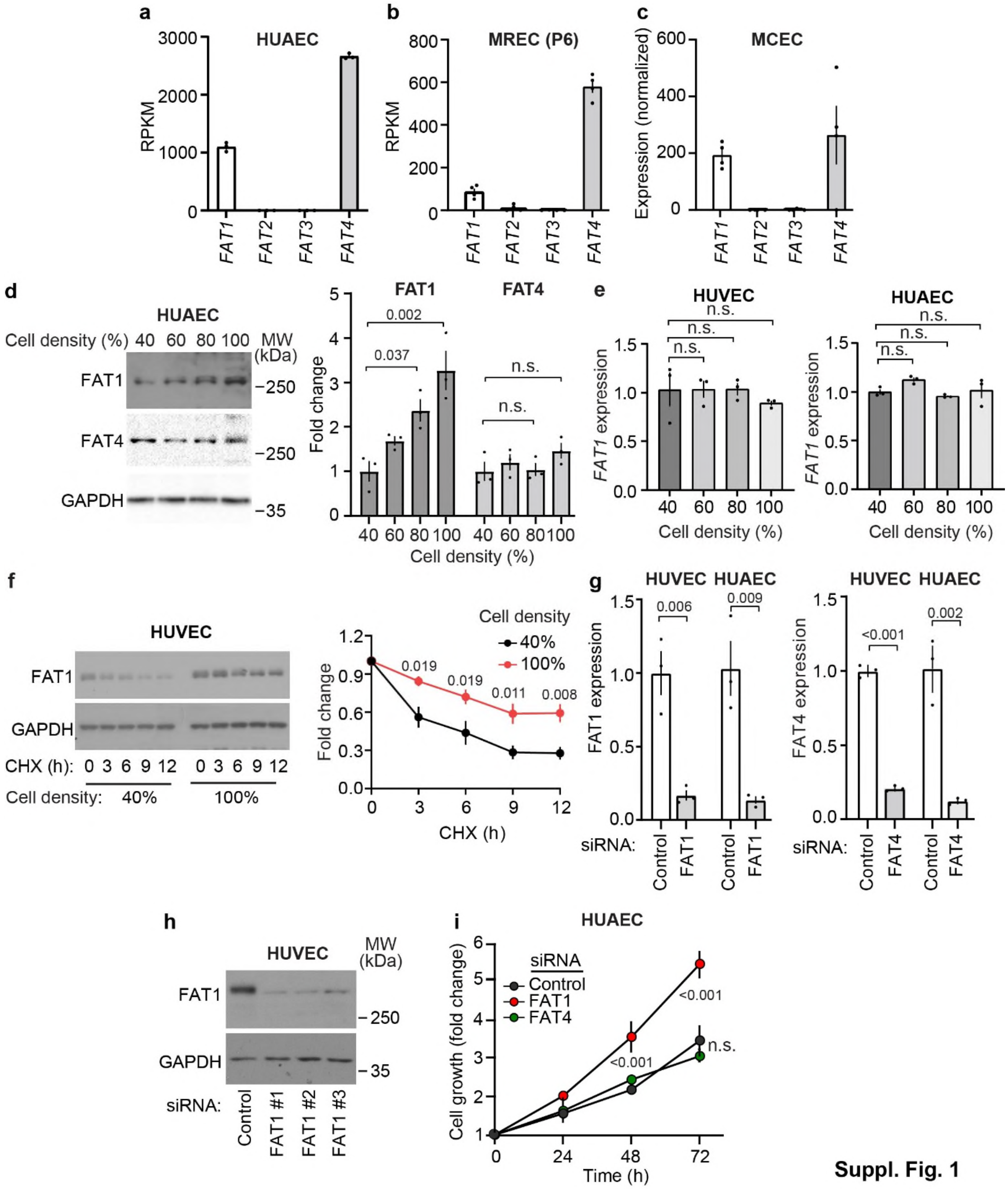

Suppl. Fig. 1

Suppl. Fig. 1. Loss of endothelial FAT1 increase cell proliferation. (**a**) RNA prepared from HUAECs was analyzed by RNA sequencing to determine expression of

genes. Shown are the results for FAT1-4 (n=3 independently performed experiments). (**b,c**) Analysis of FAT1-4 expression by RNA-sequencing in murine retinal endothelial cells (MREC) isolated at postnatal day 6 (Accession Number: GSE199858 [https://www.ncbi.nlm.nih.gov/geo/query/acc.cgi?acc=GSE199858]) (b; n=4 independently performed experiments) and in adult murine cardiac endothelial cell (MCEC) (Accession Number: GSE180794/GSE180733 [https://www.ncbi.nlm.nih.gov/geo/query/acc.cgi?acc=GSE180733]) (c; n=4 independently performed experiments). (**d**) Expression of FAT1 and FAT4 in HUAECs at different cellular densities as indicated. Shown is a representative immunoblot and the statistical evaluation (n=3 independently performed experiments). GAPDH served as a loading control. (**e**) Relative *Fat1* mRNA levels in HUVECs and HUAECs at different cellular densities (n=3 independently performed experiment). (**f**) FAT1 protein turnover in HUVECs kept at 40% density or at confluence (100% density) as determined by incubation of cells with 50 µg/ml of cycloheximide (CHX) for the indicated time periods. Shown is a representative immunoblot with GAPDH loading control and the statistical evaluation (n=3 independent experiments). (**g**,**h**) Knock-down efficiency of siRNA against human FAT1 and FAT4 in HUVECs and HUAECs determined by qRT-PCR (g, n=3 independently performed experiments) or Western blotting (h). (**i**) Cell growth of HUAECs after siRNA-mediated knock-down of FAT1 and FAT4 as determined using a colorimetric cell proliferation assay (n=4 independently performed experiments). Shown are mean values ± SEM. n.s., non-significant. Comparisons were performed using one-was ANOVA and Tukey's post-hoc test (d,e), unpaired t-test (g) or two-way ANOVA and Bonferroni's post-hoc test (f,i).

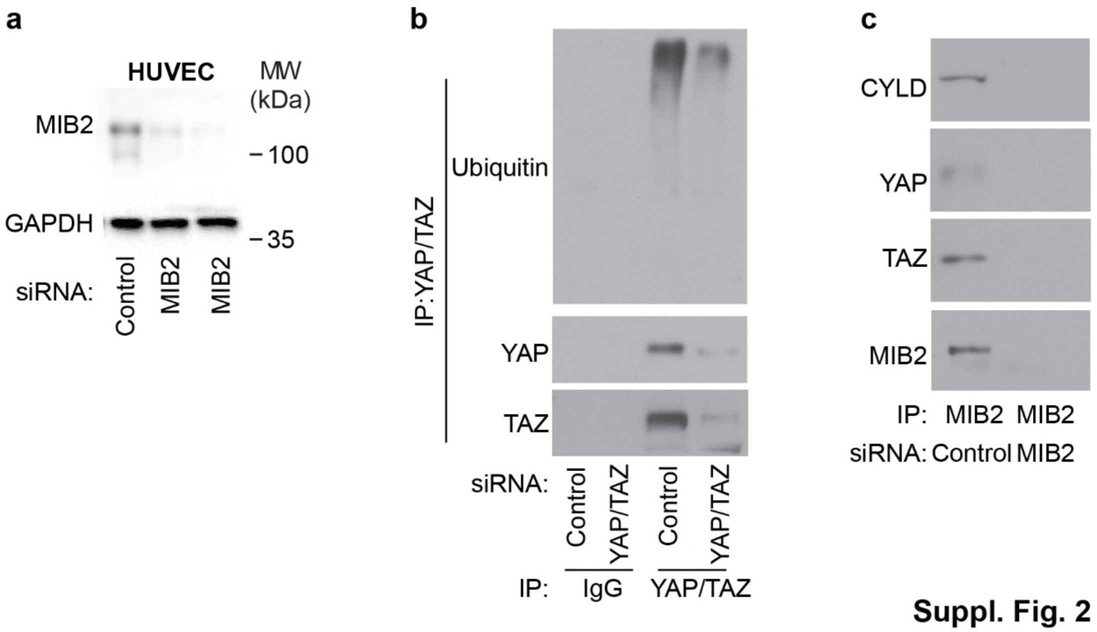

Suppl. Fig. 2

Suppl. Fig. 2. MIB2 knock-down efficiency. (**a**) HUVECs were transfected with control siRNA or siRNA directed against *MIB2*. 36 hours later, cells were lyzed, and MIB2 protein levels were determined by immunoblotting using an anti-MIB2 antibody. GAPDH served as a loading control. (**b**,**c**) HUVECs were transfected with control siRNA or siRNA directed against *YAP/TAZ* (b) and *MIB2* (c). Thereafter, cells were lysed, and YAP/TAZ (b) and MIB2 (c) were immunoprecipitated. Immunoprecipitates were analyzed by Western blotting with the indicated antibodies. Shown is a representative of 3 independently performed experiments. In b, additional controls using an unspecific IgG were performed as indicated.

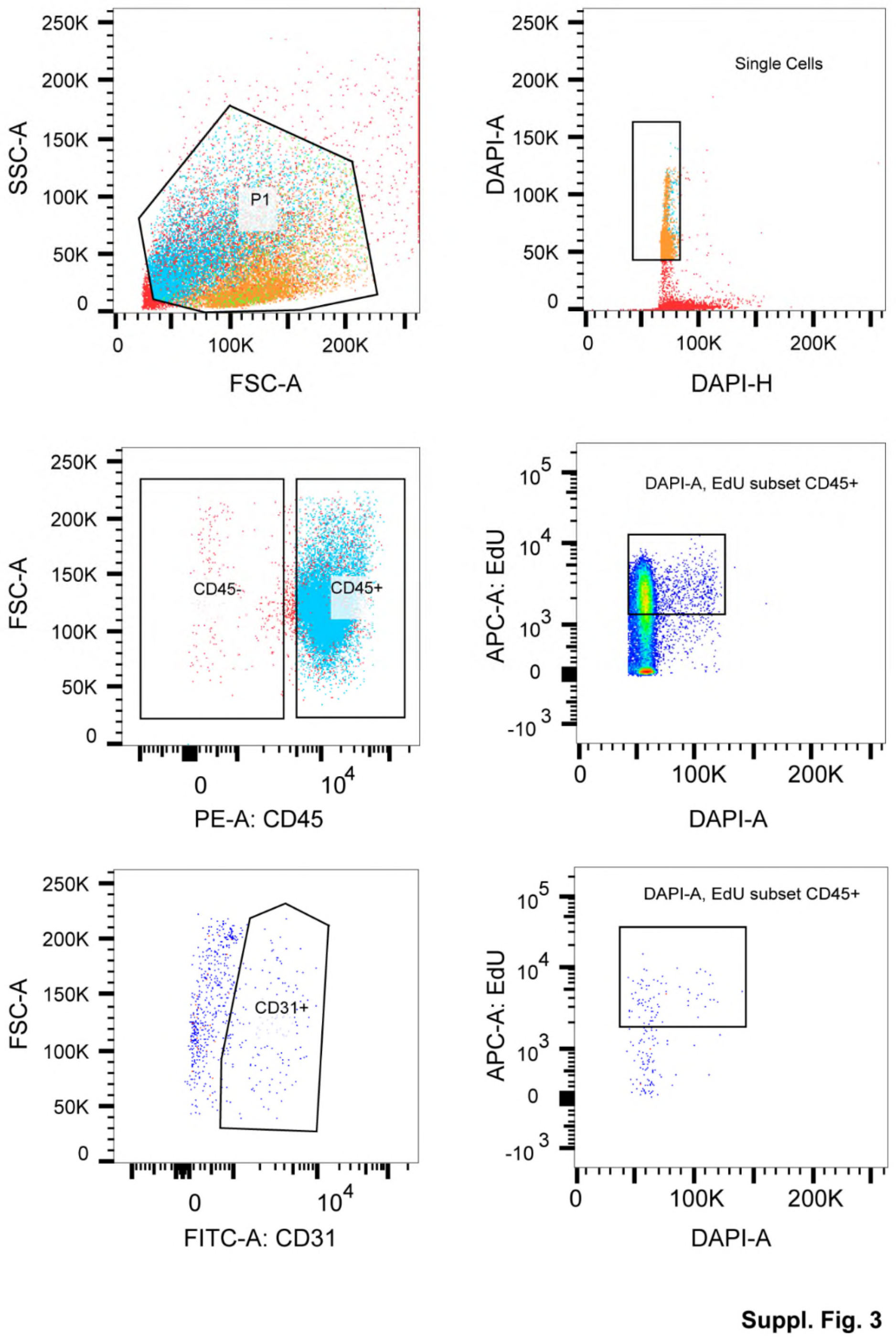

Suppl. Fig. 3. Gating strategy for the analysis or sorting of total or proliferating endothelial cell populations in primary tumors. Tumors were digested and strained through 70 µm and 40 µm filters. Afterwards, cells were stained with anti CD31-FITC and anti-CD45-PE antibodies (or with anti-CD31-PE and anti-CD45-FITC antibodies) followed by EdU-AF 647 and DAPI staining.

Supplementary Table

Gene names	Mean_Fat	Mean_Ctrl	Coeffect	padjust	Rank
ATAD3B	26,54110126	0	26,5411013	3,32E-08	1
MIB2	24,21891038	0	24,2189104	3,42E-08	2
SAP18	25,84850533	0	25,8485053	3,67E-08	3
FAT1	30,97390716	0	30,9739072	3,76E-08	4
EMC10	25,16599518	0	25,1659952	4,32E-08	5
IL2RA	31,66403917	0	31,6640392	4,89E-08	6
TIMMDC1	24,65114728	0	24,6511473	5,49E-08	7
POLRMT	24,40673507	0	24,4067351	5,66E-08	8
B3GALT6	23,19809716	0	23,1980972	5,83E-08	9
CD46	22,57114607	0	22,5711461	6,51E-08	10
NUP188	22,49699903	0	22,496999	6,65E-08	11
TMEM57	24,48642709	0	24,4864271	6,71E-08	12
QSOX1	23,24117579	0	23,2411758	6,77E-08	13
C3orf39;POMGNT2	24,18134815	0	24,1813482	6,81E-08	14
ACAD8	21,92120676	0	21,9212068	7,58E-08	15
TIMM21;C18orf55	24,84163009	0	24,8416301	7,62E-08	16
TSR1	23,41617774	0	23,4161777	7,73E-08	17
RAD21	21,66437182	0	21,6643718	7,79E-08	18
NOTCH1	22,78314178	0	22,7831418	7,82E-08	19
ATR	23,10487823	0	23,1048782	8,68E-08	20
NCAPD3	26,34410271	0	26,3441027	8,83E-08	21
POLR1D	23,38587343	0	23,3858734	9,04E-08	22
LTN1	21,95068778	0	21,9506878	9,13E-08	23
NEURL4;hCG_42028	24,18274096	0	24,182741	9,87E-08	24
NCAPG2	26,78002582	0	26,7800258	1,02E-07	25
MAPK6	21,36494345	0	21,3649435	1,34E-07	26
KNS2	25,15546547	0	25,1554655	1,59E-07	27
NCAPH2	25,80907927	0	25,8090793	1,63E-07	28
ACAD11	25,05643207	0	25,0564321	3,30E-07	29
CRISPLD1	22,40567296	0	22,405673	3,46E-07	30
PWP1	24,01607153	0	24,0160715	7,79E-07	31
TMCC3	22,9851706	0	22,9851706	8,61E-07	32
ATAD3A	32,91149197	27,22373117	5,68776081	5,07E-05	33
DOCK4	27,32261168	18,60792046	8,71469123	6,42E-05	34
TANC1	29,25602619	24,4481291	4,8078971	3,17E-04	35
KLC2	29,99222501	23,16844631	6,82377871	3,36E-04	36
HEL-S-61;	33,92053947	30,45759779	3,46294168	3,59E-04	37
TYK2	26,57260414	20,14763619	6,42496796	4,57E-04	38
KNS2	31,36648576	28,18798394	3,17850183	7,70E-04	39
PGAM5	30,91705431	26,99671279	3,92034153	8,23E-04	40
ABCD3	27,02266945	23,33782585	3,6848436	1,55E-03	41
KLC4	26,7992701	24,25401963	2,54525048	4,31E-03	42
SMC4	30,005991	25,01531488	4,99067612	4,83E-03	43
KEAP1	24,17473734	21,37420692	2,80053042	6,38E-03	44
RPRC1;MAP7D1	25,37467468	23,46853206	1,90614262	6,72E-03	45
CRIM1	27,65389325	25,22361381	2,43027944	1,29E-02	46
VAPB	30,32392575	28,85846341	1,46546234	1,83E-02	47
TBL2	25,9734743	24,61305554	1,36041876	1,91E-02	48
SLC25A11	28,34882285	27,03115013	1,31767272	2,29E-02	49
DNAJB6	25,58767712	24,39842515	1,18925197	2,71E-02	50
DNAJB12	27,16108324	26,02887605	1,13220719	2,90E-02	51
WDR26	25,03871093	23,88573624	1,15297469	2,92E-02	52
PLD1	26,38268931	21,80103145	4,58165787	2,99E-02	53
ACVRL1	24,37393643	23,16197316	1,21196328	3,14E-02	54
CHPF2	24,69495341	23,54754968	1,14740374	3,60E-02	55
SLC25A5	29,6131615	28,56620969	1,04695181	4,12E-02	56

Suppl. Tab. 1. Proteins enriched by immunoprecipitation of FAT^{ICD} from HUVEC lysate (s. Fig. 6c). Shown are statistically post-processed proteomics data using two-

side Bayesian moderated t-test provided by the limma package and p values were adjusted for multiple hypothesis testing using the method by Benjamini-Hochberg.
