# Supplementary material for: Endothelial FAT1 inhibits angiogenesis by controlling YAP/TAZ protein degradation via E3 ligase MIB2
Source: Nat Commun. 2023 Apr 8;14:1980. doi: 10.1038/s41467-023-37671-x (PMC10082778; doi:10.1038/s41467-023-37671-x)
Supplement: Supplementary file 2 — Reporting Summary [file 41467_2023_37671_MOESM2_ESM.pdf]

nature portfolio

Corresponding author(s): Stefan Offermanns

Last updated by author(s): 21.03.2023

Reporting Summary

Nature Portfolio wishes to improve the reproducibility of the work that we publish. This form provides structure for consistency and transparency in reporting. For further information on Nature Portfolio policies, see our Editorial Policies and the Editorial Policy Checklist.

Statistics

For all statistical analyses, confirm that the following items are present in the figure legend, table legend, main text, or Methods section.

n/a	Confirmed	
☐	☒	The exact sample size (*n*) for each experimental group/condition, given as a discrete number and unit of measurement
☐	☒	A statement on whether measurements were taken from distinct samples or whether the same sample was measured repeatedly
☐	☒	The statistical test(s) used AND whether they are one- or two-sided<br>*Only common tests should be described solely by name; describe more complex techniques in the Methods section.*
☐	☒	A description of all covariates tested
☐	☒	A description of any assumptions or corrections, such as tests of normality and adjustment for multiple comparisons
☐	☒	A full description of the statistical parameters including central tendency (e.g. means) or other basic estimates (e.g. regression coefficient) AND variation (e.g. standard deviation) or associated estimates of uncertainty (e.g. confidence intervals)
☐	☒	For null hypothesis testing, the test statistic (e.g. *F*, *t*, *r*) with confidence intervals, effect sizes, degrees of freedom and *P* value noted<br>*Give P values as exact values whenever suitable.*
☒	☐	For Bayesian analysis, information on the choice of priors and Markov chain Monte Carlo settings
☒	☐	For hierarchical and complex designs, identification of the appropriate level for tests and full reporting of outcomes
☒	☐	Estimates of effect sizes (e.g. Cohen's *d*, Pearson's *r*), indicating how they were calculated

Our web collection on statistics for biologists contains articles on many of the points above.

Software and code

Policy information about availability of computer code

Data collection	qRT-PCR: LightCycler 48 0 software l.5.0SP3<br>imaging, histology analysis: Leica-SP5 FLIM, Olympus cell AM software, IncuCyte®ZOOM life cell imaging system version 20181.16628.28170<br>spectrophotometry: Skanlt RE for MSS 2.4.2<br>FACS Canto II
Data analysis	statistics and graphs: GraphPad Prism software 9.3.1<br>image analysis: ImageJ/FIJI 1.53q, Leica LAS-AF Lite version 4.3<br>MRI data:Medis Suite Qmass digital imaging software version 4.0.56.6<br>FlowJo.V 10.6.1

For manuscripts utilizing custom algorithms or software that are central to the research but not yet described in published literature, software must be made available to editors and reviewers. We strongly encourage code deposition in a community repository (e.g. GitHub). See the Nature Portfolio guidelines for submitting code & software for further information.

Data

Policy information about availability of data

All manuscripts must include a data availability statement. This statement should provide the following information, where applicable:

- Accession codes, unique identifiers, or web links for publicly available datasets
- A description of any restrictions on data availability
- For clinical datasets or third party data, please ensure that the statement adheres to our policy

The source data underlying Figures 1a-e, 2b-c, 2e-f, 2h-j, 3b, 3d-h, 4a- 4d, 5a-f, 6a-b, 6d-i 7a-7f, 8a-8d and Supplementary Figures 1a-i and 2a-c are provided as source data file.
The mass spectrometric data generated in this study have been deposited in the ProteomeXchange Consortium via the MASSIVE partner repository [10.1093/nar/gkz984] under the accession code PXD040114.

Human research participants

Policy information about studies involving human research participants and Sex and Gender in Research.

Reporting on sex and gender	N/A
Population characteristics	*Describe the covariate-relevant population characteristics of the human research participants (e.g. age, genotypic information, past and current diagnosis and treatment categories). If you filled out the behavioural & social sciences study design questions and have nothing to add here, write "See above."*
Recruitment	*Describe how participants were recruited. Outline any potential self-selection bias or other biases that may be present and how these are likely to impact results.*
Ethics oversight	*Identify the organization(s) that approved the study protocol.*

Note that full information on the approval of the study protocol must also be provided in the manuscript.

Field-specific reporting

Please select the one below that is the best fit for your research. If you are not sure, read the appropriate sections before making your selection.

☒ Life sciences ☐ Behavioural & social sciences ☐ Ecological, evolutionary & environmental sciences

For a reference copy of the document with all sections, see nature.com/documents/nr-reporting-summary-flat.pdf

Life sciences study design

All studies must disclose on these points even when the disclosure is negative.

Sample size	Sample size was determined on basis of trial experiments or experiments done previously.
Data exclusions	Samples were excluded in cases where cDNA quality or tissue quality after processing was poor (below commonly accepted standards). Animals were excluded from experiments if they showed any signs of sickness (weight loss more than 20 %, skin infection, shaggy fur, loss of / or reduced movements, abnormal breathing).
Replication	Number of independent experiments are given in the Figure legends. Each experiment was repeated at least twice under independent conditions.
Randomization	No randomization was used for samples. In animal experiments, mice were caged with blinded cage numbers and random orders.
Blinding	The investigator was blinded to the group allocation and during the experiment. In animal experiments, mice were caged with blinded cage numbers and random orders.

Reporting for specific materials, systems and methods

We require information from authors about some types of materials, experimental systems and methods used in many studies. Here, indicate whether each material, system or method listed is relevant to your study. If you are not sure if a list item applies to your research, read the appropriate section before selecting a response.

Materials & experimental systems

n/a	Involved in the study
☐	☒ Antibodies
☐	☒ Eukaryotic cell lines
☒	☐ Palaeontology and archaeology
☐	☒ Animals and other organisms
☒	☐ Clinical data
☒	☐ Dual use research of concern

Methods

n/a	Involved in the study
☒	☐ ChIP-seq
☐	☒ Flow cytometry
☒	☐ MRI-based neuroimaging

Antibodies

Antibodies used

For Western Blot, the following antibodies were used: FAT1 (Rabbit, Abcam, catalog# ab109202,1:1000), FAT4 (Rabbit, Abcam, catalog# ab130076,1:200), HA (Mouse, Sigma-Aldrich, catalog #H6533, 1:2000), Flag M2 (Mouse, Sigma-Aldrich, catalog #A8592) YAP/TAZ. (Rabbit, Cell Signaling, catalog #8418, 1:1000), YAP (Rabbit, Cell Signaling, catalog #4912, 1:500), GAPDH (Rabbit, Cell Signaling, catalog #2118, 1:2000), LATS1 (Rabbit, Cell Signaling, catalog #3477, 1:1000), phospho-LATS1 (S909) (Rabbit, Cell Signaling, catalog #9157), phospho-LATS1 (1079) (Rabbit, Cell Signaling, catalog #8654, 1:1000), phospho-MST1 (T183) (Rabbit, Cell Signaling, catalog #3681, 1:1000), phospho-MOBl (T35) (Rabbit, Cell Signaling, catalog #8699, 1:1000), Ubiquitin (Rabbit, Cell Signaling, catalog #3936S, 1:1000), CYLD (Rabbit, Cell Signaling, catalog #8462, 1:1000), MIB2 (Rabbit, Bethyl Laboratories, catalog #A301-414A, 1:1000). The horseradish peroxidase-conjugated antibodies directed against Rabbit or Mouse lgG (Cells Signaling, catalog #7074 and 7076, 1:3000) or veriblot for IP detection reagent (ab131366) were used. For lmmunofluorescence staining, the following antibodies were used: lsolectin GS-IB4 (Thermo Fisher Scientific, catalog# 121413/132450, 1:500), ERG (Rabbit, abeam, catalog# ab92513, 1:100), Cleaved Caspase-3 (Rabbit, Cell Signaling, catalog #9664, 1:100), CD31 (Rat, Abeam, catalog #ab 7388, 1:100). The following donkey anti-Rat/Rabbit IgG highly cross-adsorbed secondary antibodies AlexaFluorTM-488- or AlexaFluorTM-594 (Thermo Fisher Scientific, catalog #A21208, #A21206, #A21207, #A21209, 1:200) were used. For FACS analysis and sorting, the following antibodies were used: CD31-FITC (BD Bioscience, catalog #558738, 1:100)/ CD31-PE (eBioscience, catalog #12-0311-82, 1:100) and CD45-PE (eBioscience, catalog #12-0451-82 1:100) / CD45-FITC (BD Bioscience, catalog #553079, 1:100)

Validation

Western blot antibodies were validated according to instruction on the manufactures' website or in eukaryotic cell lines after gene silencing.
For immunofluorescence staining, antibodies were validated using negative group of isotype matched control and omission of primary antibody control to identify background staining.
Antibodies for flow cytometry were specifically validated using isotype matched control combined with identification of gene expression pattern in sorted cells by qRT-PCR.

Eukaryotic cell lines

Policy information about cell lines and Sex and Gender in Research

Cell line source(s)

HUVEC were obtained from Lonza (C2519A,) or Gibco (C-015-5C), HUAEC was purchased from Provitro AG (1210112) HEK293 cell was obtained from ATCC.

Authentication

No further authentication.

Mycoplasma contamination

Tested negative.

Commonly misidentified lines
(See ICLAC register)

No commonly misidentified lines were used.

Animals and other research organisms

Policy information about studies involving animals; ARRIVE guidelines recommended for reporting animal research, and Sex and Gender in Research

Laboratory animals

Stated in the method section under "animal models".
Mice carrying a floxed allele of Fat1 were described before (Caruso, N. et al. 2013). Generation of mice with floxed alleles of Yap and Wwtr1 (Taz) has been described in previous publication (Wang, L. et al. 2020). Mice carrying a knock-out first allele of the Mib2 gene (Mib2tm1a(EUCOMM)Wtsi/IcsOrl) were obtained from The European Mouse Mutant Archive. To generate a floxed Mib2 allele, mice were crossed with ACTB-FLPe mice (The Jackson Laboratory, strain #005703) 65. Mice were crossed with Tek-CreERT2 mice (Korhonen, H et al. 2009) to obtain animals with inducible endothelium specific deficiency.
All above mice were backcrossed onto a C57BL/6J background at least 8 to 10 times. Both male and female were used for all experiments. Littermates lacking Cre or the floxed alle and treated with tamoxifen in the same way were used as controls.
Postnatal angiogenesis experiments were started at postnatal day P1 and ended at postnatal day P6 as described in the method section.
Experiments of tumor angiogenesis were started by tamoxifen injection at an animal age of 13-15 weeks and animals were sacrificed at 18-20 weeks.
Hind-limb ischemia experiments were started by tamoxifen injection when mice were 11-13 weeks old and were ended when mice were 15-17 weeks old.

	Myocardial infarction experiments were started when mice were 8-10 weeks old and were ended when mice were 16-18 weeks old. Mice were housed under a 12-hour light-dark cycle with free access to food and water and under specific pathogen-free conditions.
Wild animals	Not involved.
Reporting on sex	Regarding the sex of the animals used in the study, we used both females and males. For in vitro study, we used human umbilical vein and artery endothelial cells, which are derived from the embryo, whose sex remained unknown to us. Thus, sex was not considered in the in vitro experiments with cells.
Field-collected samples	Not invovled.
Ethics oversight	Animal experiments were approved by the Institutional Animal Care and Use Committee of the Regierungsprasidium Darmstadt and in accord with Directive 2010/63/EU of the European Parliament on the protection of animals used for scientific purposes.

Note that full information on the approval of the study protocol must also be provided in the manuscript.

Flow Cytometry

Plots

Confirm that:

☒ The axis labels state the marker and fluorochrome used (e.g. CD4-FITC).

☒ The axis scales are clearly visible. Include numbers along axes only for bottom left plot of group (a 'group' is an analysis of identical markers).

☐ All plots are contour plots with outliers or pseudocolor plots.

☒ A numerical value for number of cells or percentage (with statistics) is provided.

Methodology

Sample preparation	Primary tumors were harvested, minced, filtered and then stained with anti-CD31 and anti-CD45 antibodies as described in the method section under "Cell sorting and Flow cytometry analysis".
Instrument	FACS CantoII
Software	FACS DIVA v6.l.2
Cell population abundance	Not applicable.
Gating strategy	The gating strategies are depicted in Supplementary lnformation (Supplementray Figure 3).

☒ Tick this box to confirm that a figure exemplifying the gating strategy is provided in the Supplementary Information.
